# Fibrin(ogen) engagement of *S. aureus* promotes the host antimicrobial response and suppression of microbe dissemination following peritoneal infection

**DOI:** 10.1371/journal.ppat.1010227

**Published:** 2022-01-18

**Authors:** Oscar Negrón, Woosuk S. Hur, Joni Prasad, David S. Paul, Sarah E. Rowe, Jay L. Degen, Sara R. Abrahams, Silvio Antoniak, Brian P. Conlon, Wolfgang Bergmeier, Magnus Hӧӧk, Matthew J. Flick

**Affiliations:** 1 Department of Pathology and Laboratory Medicine, UNC Blood Research Center, Lineberger Comprehensive Cancer Center, University of North Carolina at Chapel Hill, Chapel Hill, North Carolina, United States of America; 2 Division of Experimental Hematology, Cincinnati Children’s Hospital Medical Center and University of Cincinnati School of Medicine, Cincinnati, Ohio, United States of America; 3 Department of Biochemistry, UNC Blood Research Center, University of North Carolina at Chapel Hill, Chapel Hill, North Carolina, United States of America; 4 Department of Microbiology and Immunology, University of North Carolina at Chapel Hill, Chapel Hill, North Carolina, United States of America; 5 Center of Infectious and Inflammatory Diseases, Texas A&M Health Sciences Center, Houston, Texas, United States of America; Columbia University, UNITED STATES

## Abstract

The blood-clotting protein fibrin(ogen) plays a critical role in host defense against invading pathogens, particularly against peritoneal infection by the Gram-positive microbe *Staphylococcus aureus*. Here, we tested the hypothesis that direct binding between fibrin(ogen) and *S*. *aureus* is a component of the primary host antimicrobial response mechanism and prevention of secondary microbe dissemination from the peritoneal cavity. To establish a model system, we showed that fibrinogen isolated from Fibγ^Δ5^ mice, which express a mutant form lacking the final 5 amino acids of the fibrinogen γ chain (termed fibrinogenγ^Δ5^), did not support *S*. *aureus* adherence when immobilized and clumping when in suspension. In contrast, purified wildtype fibrinogen supported robust adhesion and clumping that was largely dependent on *S*. *aureus* expression of the receptor clumping factor A (ClfA). Following peritoneal infection with *S*. *aureus* USA300, Fibγ^Δ5^ mice displayed worse survival compared to WT mice coupled to reduced bacterial killing within the peritoneal cavity and increased dissemination of the microbes into circulation and distant organs. The failure of acute bacterial killing, but not enhanced dissemination, was partially recapitulated by mice infected with *S*. *aureus* USA300 lacking ClfA. Fibrin polymer formation and coagulation transglutaminase Factor XIII each contributed to killing of the microbes within the peritoneal cavity, but only elimination of polymer formation enhanced systemic dissemination. Host macrophage depletion or selective elimination of the fibrin(ogen) β2-integrin binding motif both compromised local bacterial killing and enhanced *S*. *aureus* systemic dissemination, suggesting fibrin polymer formation in and of itself was not sufficient to retain *S*. *aureus* within the peritoneal cavity. Collectively, these findings suggest that following peritoneal infection, the binding of *S*. *aureus* to stabilized fibrin matrices promotes a local, macrophage-mediated antimicrobial response essential for prevention of microbe dissemination and downstream host mortality.

## Author summary

The Gram-positive bacterium *Staphylococcus aureus* (*S*. *aureus*) produces a number of soluble and surface-associated proteins that bind the host coagulation protein fibrinogen. The contribution of fibrinogen-*S*. *aureus* binding through the fibrinogen receptor clumping factor A (ClfA) in peritoneal infection has not been defined. Elimination of the binding motif on fibrinogen for ClfA or deletion of ClfA from *S*. *aureus* significantly reduced *S*. *aureus-*fibrinogen binding and bacterial clumping in solution. In a mouse model of peritonitis, loss of these activities resulted in diminished bacterial killing, increased bacterial dissemination, and worsened host survival. Although fibrin polymer formation and fibrin(ogen)-macrophage binding are mechanistically linked to the local antimicrobial response, fibrin formation in and of itself is not sufficient to suppress microbe dissemination. These discoveries have identified important components of the fibrin(ogen)-dependent host antimicrobial response against *S*. *aureus*, providing further understanding of this physiological response to infection which could uncover potential therapeutic strategies for peritonitis patients.

## Introduction

*Staphylococcus aureus* (*S*. *aureus*) is a common, Gram-positive bacterium that colonizes 20–80% of healthy adults [1]. It is the causative agent for a variety of illnesses ranging from minor skin infections to more serious and life-threatening conditions such as bacteremia, sepsis, infective endocarditis, and pneumonia [2]. *S*. *aureus* is frequently identified in both community- and hospital-acquired settings [3]. Even with appropriate treatment strategies, *S*. *aureus* bacteremia has a 30-day mortality rate of 20–40% [4], an issue that is further complicated by the emergence of methicillin and vancomycin resistant (M.R.S.A. and V.R.S.A.) strains of *S*. *aureus* [5]. Thus, there is a need to better understand the mechanisms of pathogenesis of *S*. *aureus* in order to develop improved treatment strategies that do not strictly rely on antibiotics.

*S*. *aureus* expresses multiple virulence factors that allow the bacterium to engage and manipulate components of the host coagulation system, including a variety of proteins that directly bind fibrin(ogen). Specifically, *S*. *aureus* produces a family of virulence factors called microbial surface components recognizing adhesive matrix molecules (MSCRAMMs), such as the fibrin(ogen) binding protein Clumping factor A (ClfA). ClfA binds to fibrin(ogen) at the carboxy-terminal domain of the fibrinogen γ chain and mediates bacterial clumping in host plasma and bacterial adhesion to fibrin(ogen)-coated surfaces [6]. ClfA has been identified as an important factor of *S*. *aureus* virulence in animal models of bacteremia, septic arthritis and endocarditis through fibrin(ogen)-dependent and independent mechanisms [7–11].

Although *S*. *aureus* utilizes host hemostatic factors like fibrin(ogen) to support its virulence, the host hemostatic system plays an important role in antimicrobial defense. Indeed, fibrin(ogen) can function as a protective barrier, trapping invading microbes in matrices to inhibit bacterial growth and dissemination through the host [6,12,13]. Fibrin(ogen) also functions as a modulator of the host immune response. Neutrophils and macrophages expressing the integrin receptor α_M_β_2_ can bind fibrin(ogen), leading to activation and initiation of antimicrobial functions like phagocytosis, production of reactive oxygen species (ROS), and pro-inflammatory cytokine production [14–18]. For peritoneal *S*. *aureus* infections, fibrin(ogen) was shown to play a pivotal role in host defense. Studies of fibrinogen-deficient (Fib^-/-^) mice revealed that these animals display poor survival and an inability to clear bacteria from the peritoneal cavity following acute infection [19]. Here, we tested the hypothesis that *S*. *aureus* binding to fibrin(ogen) via ClfA is a key feature of the antimicrobial mechanism following peritoneal infection. Further, we determined the impact of platelets, fibrin polymer formation, fibrin crosslinking, and the fibrin(ogen)-macrophage response to the dissemination of bacteria from the peritoneum and the establishment of systemic disease.

## Results

### The fibrin(ogen) γ-chain AGDV motif is required for *S. aureus* adhesion to immobilized fibrinogen and for clumping in a fibrinogen solution *in vitro*

**Fig 1:**
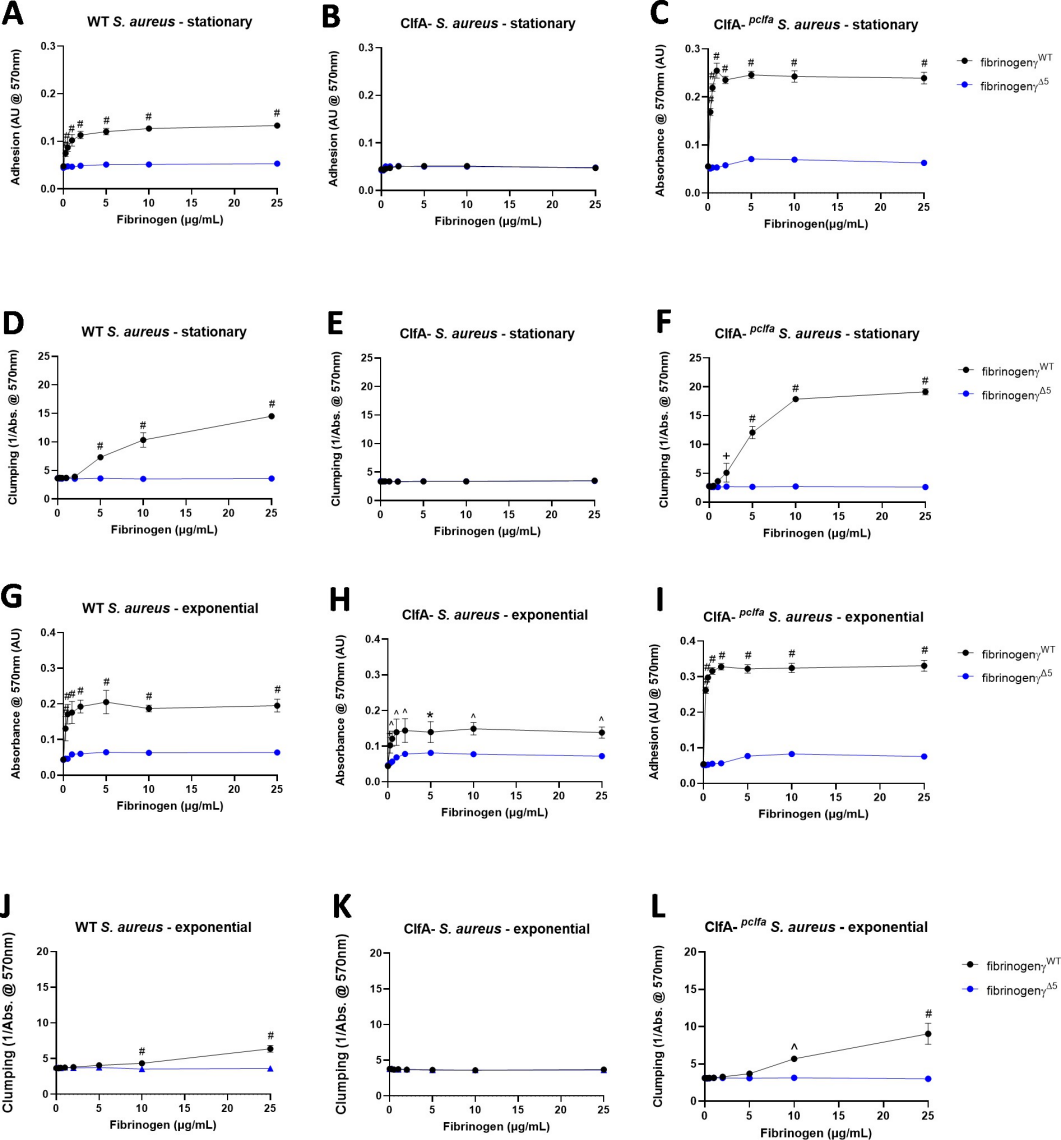
The fibrin(ogen) AGDV binding motif is required for both *S. aureus* adhesion to immobilized fibrinogen and clumping in fibrinogen solution. Adhesion of stationary phase (A) WT *S*. *aureus* USA300, (B) ClfA- *S*. *aureus* USA300, and (C) ClfA-^*pclfa*^ *S*. *aureus* USA300 to immobilized fibrinogenγ^WT^ or fibrinogenγ^Δ5^. Clumping of stationary phase (D) WT *S*. *aureus* USA300, (E) ClfA- *S*. *aureus* USA300, or (F) ClfA-^*pclfa*^ *S*. *aureus* USA300 in solutions containing fibrinogenγ^WT^ or fibrinogenγ^Δ5^. Adhesion of exponential phase (G) WT *S*. *aureus* USA300, (H) to ClfA- *S*. *aureus* USA300, or (I) ClfA-^*pclfa*^ *S*. *aureus* USA300 to immobilized fibrinogenγ^WT^ or fibrinogenγ^Δ5^. Clumping of exponential phase (J) WT *S*. *aureus* USA300, (K) ClfA- *S*. *aureus* USA300, or (L) ClfA-^*pclfa*^ *S*. *aureus* USA300 in solutions containing fibrinogenγ^WT^ or fibrinogenγ^Δ5^. Data is derived from N = 3 replicates per fibrinogen concentration per group and presented as mean absorbance (for adhesion experiments) or the inverse of mean absorbance (for clumping experiments) ± SEM. Statistical significance was determined by 2- way ANOVA with Šídák’s multiple comparisons test where + = <0.05, * = <0.01, ^ = <0.001, and # = <0.0001.

The C-terminal portion of the fibrinogen γ-chain encodes a motif for interaction of *S*. *aureus* through the receptor ClfA, as well as other *S*. *aureus* fibrinogen binding proteins such as fibronectin-binding protein A and B (Fnbp-A and Fnbp-B) [11,20–22]. Fibγ^Δ5^ mice express a mutant form of fibrinogen lacking the final AGDV motif. Adhesion and clumping experiments were performed using fibrinogen purified from wildtype (fibrinogenγ^WT^) and Fibγ^Δ5^ mice (fibrinogenγ^Δ5^) to characterize the impact of the loss of the AGDV motif on fibrinogen-*S*. *aureus* interactions. WT *S*. *aureus* USA300 grown to stationary phase bound to immobilized fibrinogenγ^WT^ in a dose dependent manner whereas fibrinogenγ^Δ5^ did not support bacterial adhesion at any coating concentration (Fig 1A). Genetic elimination of ClfA from the bacterial surface (ClfA-) resulted in loss of bacterial binding to both immobilized fibrinogen species (Fig 1B). Notably, analysis of ClfA-^*pclfa*^ *S*. *aureus*, a complemented ClfA- USA300 strain in which expression of ClfA was reconstituted, restored binding to fibrinogenγ^WT^ but not to fibrinogenγ^Δ5^ (Fig 1C). In assays evaluating solution phase *S*. *aureus* clumping, fibrinogenγ^WT^ supported clumping in a dose dependent manner but no clumping was observed with fibrinogenγ^Δ5^ (Fig 1D). As expected, ClfA- *S*. *aureus* USA300 did not support clumping with fibrinogenγ^WT^ and fibrinogenγ^Δ5^ (Fig 1E), whereas ClfA-^*pclfa*^ *S*. *aureus* USA300 displayed a clumping pattern identical to WT *S*. *aureus* USA300 (Fig 1F).

*S*. *aureus* produces other fibrinogen-binding proteins, including Fnbp-A and Fnbp-B that engage fibrin(ogen) via the fibrinogen γ-chain [6]. However, while ClfA is expressed throughout the entire growth cycle of *S*. *aureus*, Fnbp-A and Fnbp-B are expressed predominantly during the exponential growth phase [23–26]. To determine a potential role for these additional fibrinogen-binding proteins, we performed adhesion and clumping experiments with *S*. *aureus* USA300 grown to exponential phase in the presence of fibrinogenγ^WT^ or fibrinogenγ^Δ5^. Similar to *S*. *aureus* at stationary phase, exponential phase WT *S*. *aureus* USA300 bound immobilized fibrinogenγ^WT^ in a dose dependent manner whereas fibrinogenγ^Δ5^ did not support bacterial adhesion at any coating concentration (Fig 1G). However, exponential phase ClfA- *S*. *aureus* USA300 was able to bind fibrinogenγ^WT^, but fibrinogenγ^Δ5^ did not support bacterial adhesion (Fig 1H). ClfA-^*pclfa*^ *S*. *aureus* displayed a pattern identical to WT *S*. *aureus* (Fig 1I). When evaluating *S*. *aureus* clumping, exponential phase WT *S*. *aureus* USA300 showed reduced clumping in the presence of fibrinogenγ^WT^ when compared to stationary phase bacteria and no clumping was observed in the presence of fibrinogenγ^Δ5^ (Fig 1J). Exponential phase ClfA- *S*. *aureus* USA300 did not support clumping with either of the fibrinogens analyzed (Fig 1K), whereas ClfA-^*pclfa*^ *S*. *aureus* displayed a pattern akin to WT *S*. *aureus* (Fig 1L). The findings suggest that the fibrinogen γ-chain AGDV motif supports fibrinogen-*S*. *aureus* interactions, with a major fraction of the binding occurring through ClfA.

### Elimination of the γ-chain AGDV motif results in decreased survival following *S. aureus* peritonitis

**Fig 2:**
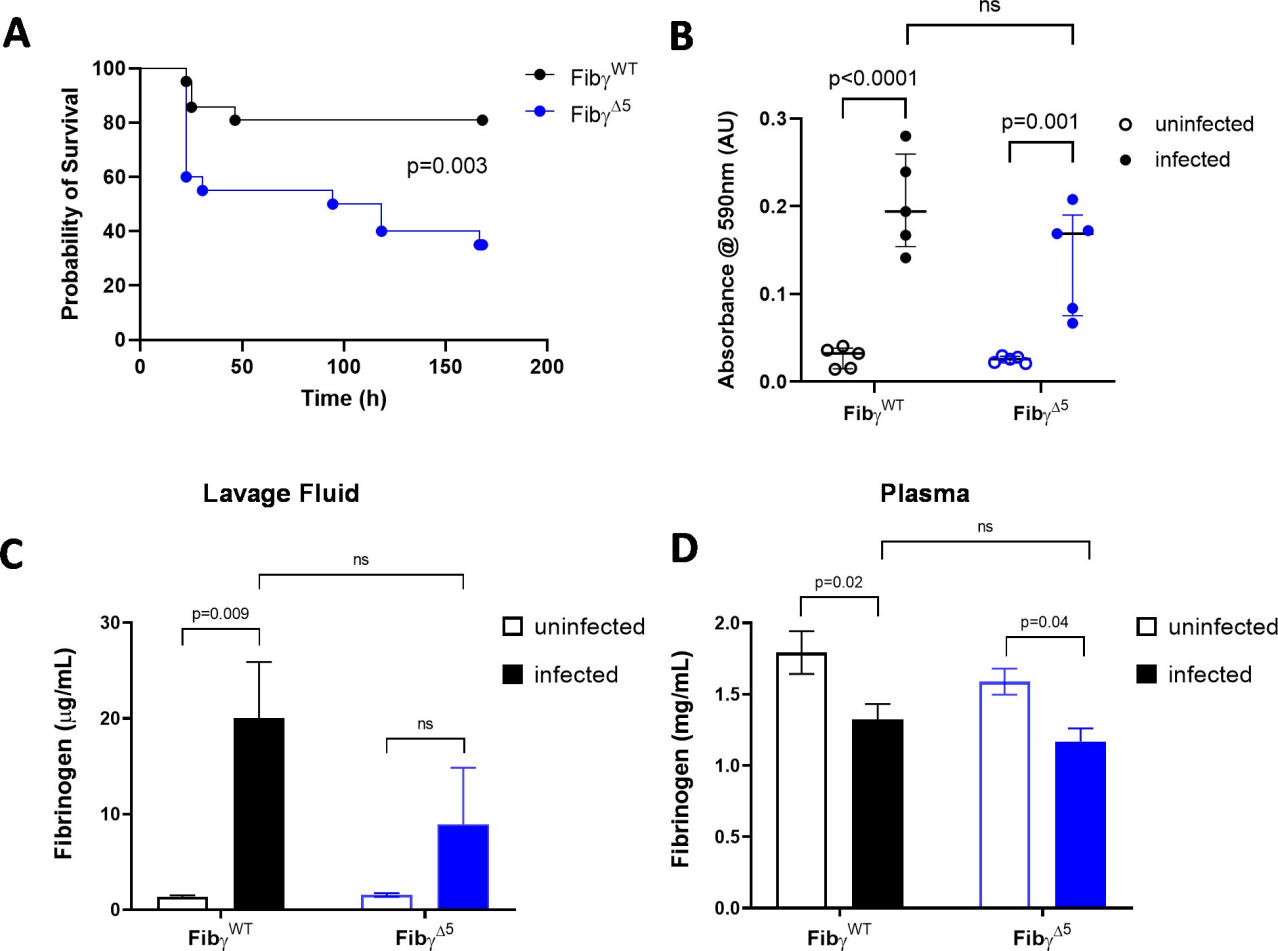
Fibγ^Δ5^ mice display decreased survival following *S. aureus* peritonitis. (A) Kaplan-Meyer log-rank analysis of Fibγ^WT^ (n = 20) and Fibγ^Δ5^ (n = 20) mice following i.p. infection with 4.7x10^8^ CFUs of *S*. *aureus* USA300. (B) Evans Blue vascular leak assay on Fibγ^WT^ and Fibγ^Δ5^ mice following i.p. injection with PBS or ~1x10^9^ CFU of *S*. *aureus* USA300. Fibrinogen ELISA on lavage fluid (C) and plasma (D) following 1 hour i.p. infection with *S*. *aureus* USA300. Data are presented as mean ± SEM and statistical significance was determined by 2-way ANOVA with Šídák’s multiple comparisons test.

To determine the role of *S*. *aureus*-fibrinogen interactions on host survival following peritoneal infection, cohorts of Fibγ^WT^ and Fibγ^Δ5^ mice were given an intraperitoneal (i.p.) infection with ~5x10^8^ CFUs of WT *S*. *aureus* USA300 and animal survival was monitored. Fibγ^Δ5^ showed a significant decrease in survival with only 40% of mice remaining after a 1-week observation period whereas Fibγ^WT^ mice were largely protected (Fig 2A). These data suggest that fibrin(ogen)-*S*. *aureus* interactions support a host defense mechanism important for host survival following peritonitis.

The accumulation of fibrinogen and other plasma components entering the peritoneal cavity following infection was next analyzed. In an Evans Blue vascular leak assay, Fibγ^WT^ and Fibγ^Δ5^ each had low levels of retrievable Evans Blue dye from the peritoneal cavity in the absence of infection (Fig 2B). In contrast, mice challenged with a peritoneal *S*. *aureus* infection had quantifiably higher levels of Evans Blue retrievable from the peritoneal cavity 1 hour after infection with no genotype-dependent differences detected (Fig 2B). ELISA assays for fibrinogen were also performed on peritoneal lavage fluid collected from Fibγ^WT^ and Fibγ^Δ5^ mice uninfected as well as 1 hour after i.p. injection with ~1x10^9^ CFUs of *S*. *aureus* USA300. Peritoneal lavage fluid from uninfected mice contained very little fibrinogen, but the lavage fluid from infected animals contained readily detectable concentrations of fibrinogen that were comparable between both genotypes (Fig 2C). Plasma fibrinogen levels were similar between both genotypes in uninfected mice as previously reported [27], and were modestly lower in each genotype 1 hour after infection (Fig 2D). Collectively, these findings indicate that the observed survival phenotypes were not due to a failure of plasma proteins, including fibrinogen, to enter the peritoneal cavity of infected Fibγ^Δ5^ mice.

### Fibγ^Δ5^ mice show increased *S. aureus* CFUs in the peritoneal cavity following infection

**Fig 3:**
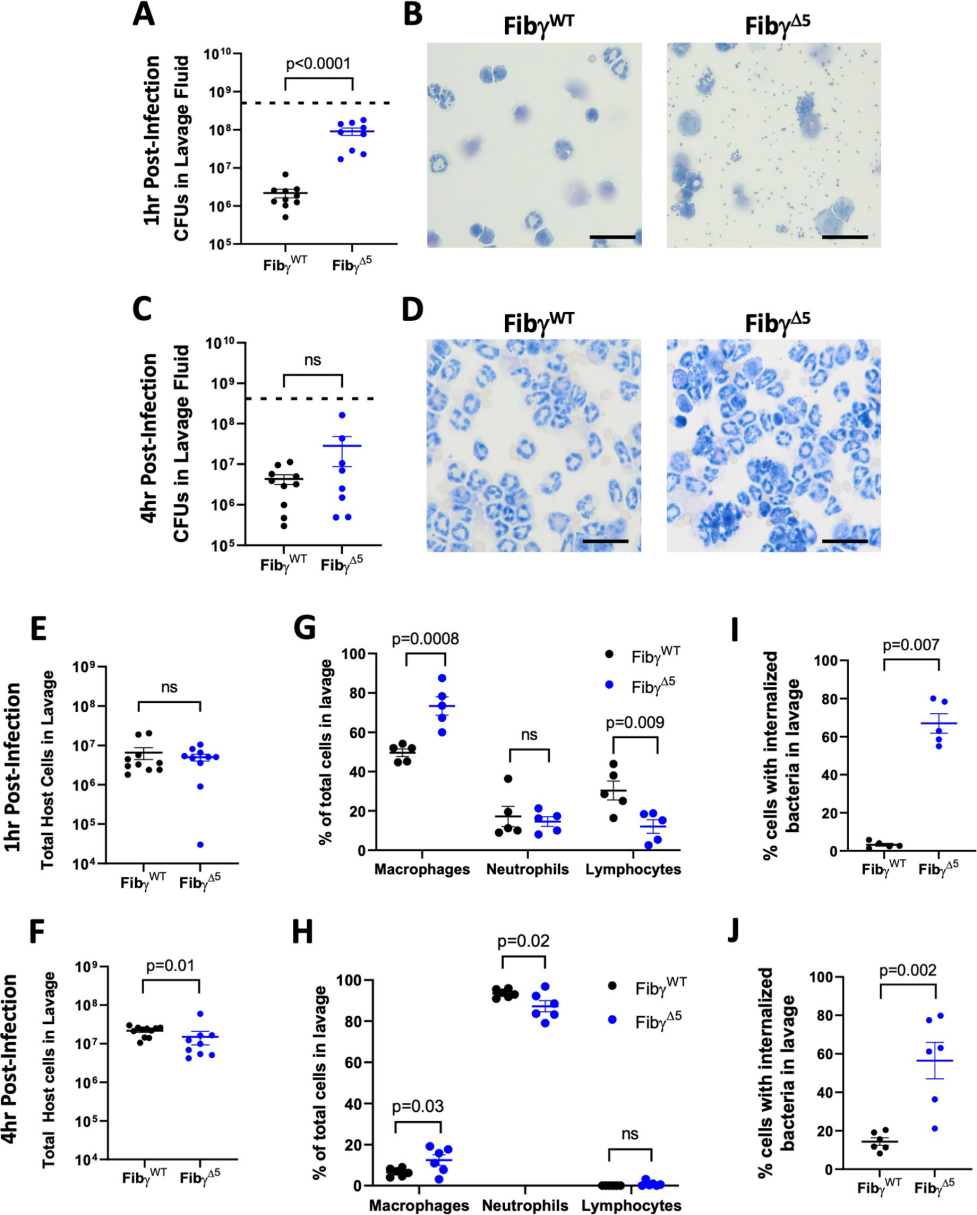
Fibγ^Δ5^ mice display increased *S. aureus* CFUs in the peritoneal cavity following infection. (A) Total bacterial CFUs in peritoneal lavage fluid of Fibγ^WT^ and Fibγ ^Δ5^ mice 1 hour after i.p. infection with 5x10^8^ CFUs of WT USA300 *S*. *aureus*. (B) Representative images of cytospin preparations of peritoneal lavage fluid collected from Fibγ^WT^ and Fibγ ^Δ5^ mice 1hr after i.p. infection with 5x10^8^ CFUs USA300 *S*. *aureus*. Note the presence of extensive free-floating bacteria in Fibγ ^Δ5^ mice compared to Fibγ^WT^. (C) Total CFU in the peritoneal lavage fluid of Fibγ^WT^ and Fibγ^Δ5^ mice 4 hours after i.p. infection with 4x10^8^ CFUs of WT USA300 *S*. *aureus*. (D) Representative images of cytospin preparations of peritoneal lavage fluid collected from Fibγ^WT^ and Fibγ^Δ5^ mice 4 hours after infection. CFU data is presented as mean ± SEM and statistical significance was determined by Mann-Whitney u-test. Dashed horizontal lines indicate the infection dose. Images were captured with 40x objective with scale bar representing 20 μm. Total cell counts from peritoneal lavage fluid 1 hour (E) and 4 hours (F) after infection with USA300 *S*. *aureus*. Data are presented as mean ± SEM and statistical significance was determined by Mann-Whitney u-test. Differential cell counts from peritoneal lavage fluid 1 hour (G) and 4 hours (H) after infection. Data are presented as mean ± SEM and statistical significance was determined by 2-way ANOVA with Šídák’s multiple comparisons test. Analysis of total cells with internalized bacteria 1 hour (I) and 4 hours (J) after infection. Data are presented as mean ± SEM and statistical significance was determined by Mann-Whitney u-test.

Previous studies identified a rapid, local fibrin-driven antimicrobial activity against *S*. *aureus* peritoneal infection [19]. To determine whether fibrinogen-*S*. *aureus* interactions are a component of that host defense mechanism, cohorts of Fibγ^WT^ and Fibγ^Δ5^ mice were challenged with an i.p. infection with ~5x10^8^ CFUs of WT *S*. *aureus* USA300 and peritoneal lavage fluid analyzed 1 or 4 hours after infection. WT mice rapidly eliminated ~99% of the initial inoculum within 1 hour in a manner similar to previous findings [19] whereas Fibγ^Δ5^ mice showed significantly higher bacterial CFUs following infection (Fig 3A). Analysis of cytospin images of the lavage fluid showed excessive free-floating bacteria in Fibγ^Δ5^ mice while Fibγ^WT^ mice had little to no free-floating bacteria (Fig 3B). Interestingly, at 4 hours after infection, no significant differences in *S*. *aureus* CFUs in the peritoneal lavage fluid were observed between genotypes (Fig 3C). Cytospin images showed little to no free-floating bacteria in the peritoneal lavage fluid of Fibγ^WT^ and Fibγ^Δ5^ mice at 4 hours after infection (Fig 3D).

No differences in total host cells retrievable from the peritoneal cavity were observed between genotypes at 1 hour (Fig 3E) post-infection; however, a modest but statistically significant decrease in cell numbers was observed in Fibγ^Δ5^ mice at 4 hours (Fig 3F) post-infection. Fibγ^Δ5^ had marginally higher macrophages and lower lymphocytes relative to Fibγ^WT^ mice within the lavage fluid at 1 hour after infection (Fig 3G). At 4 hours after infection, neutrophils were the most prevalent cell type as expected (Fig 3H), and Fibγ^Δ5^ had more macrophages but lower neutrophils relative to Fibγ^WT^ mice (Fig 3H). Quantitative assessment indicated that lavage fluid harvested from Fibγ^Δ5^ contained significantly higher numbers of host cells with internalized bacteria relative to Fibγ^WT^ mice at both 1 hour and 4 hours after infection (Figs 3I and 3J). Collectively, these data suggest that the reduction in bacterial clearance observed in Fibγ^Δ5^ mice is not due to a genotype-dependent difference in host cells within the peritoneal cavity nor a failure of the peritoneal cells to phagocytose bacteria.

### Elimination of *S. aureus*-fibrinogen γ-chain interactions results in rapid bacterial dissemination from the peritoneal cavity

**Fig 4:**
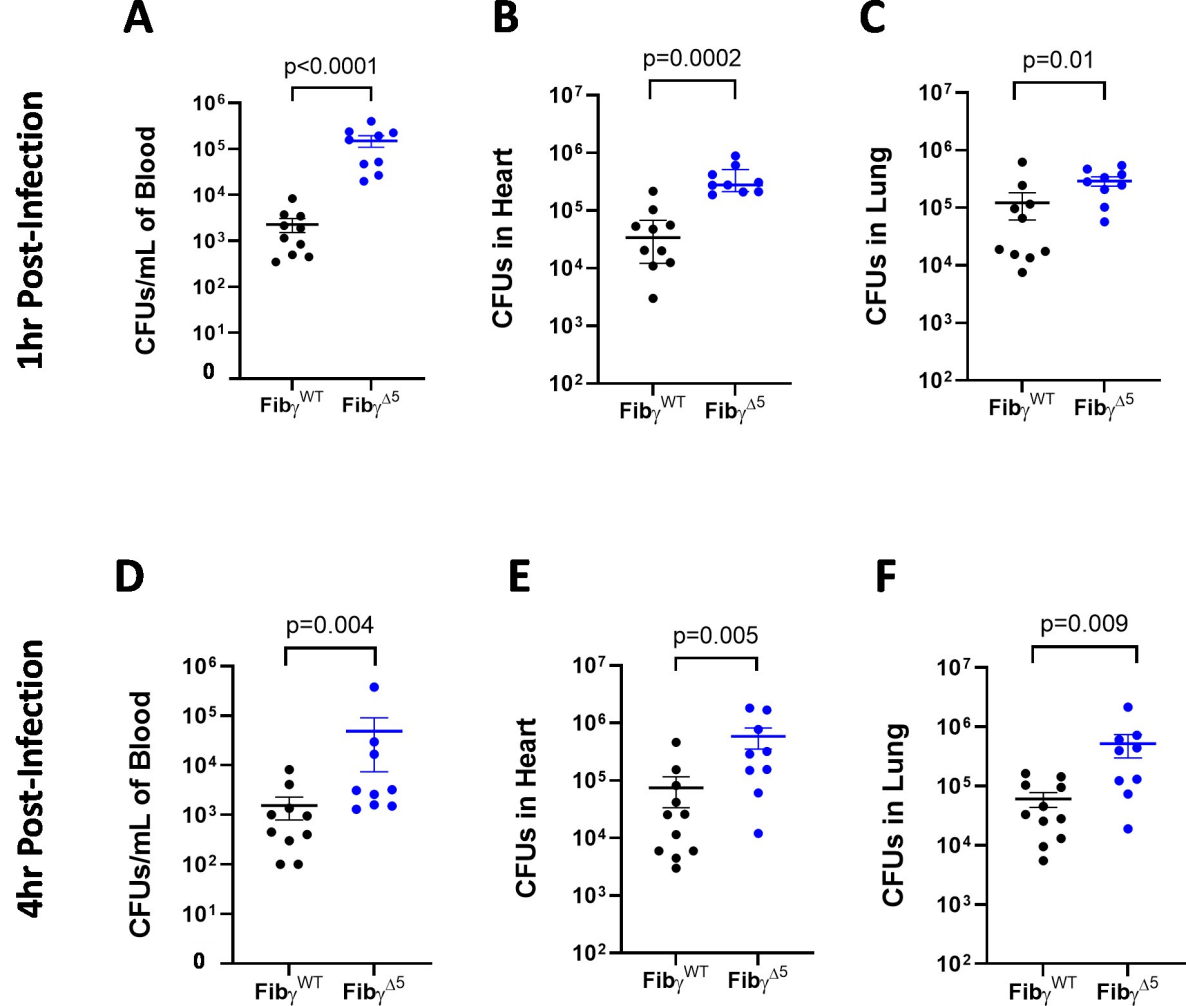
Fibγ^Δ5^ mice display increased *S. aureus* dissemination to organ tissues. Total CFUs in (A) blood, (B) heart and (C) lung of Fibγ^WT^ and Fibγ^Δ5^ mice 1 hour after i.p. infection with 5x10^8^ CFUs of WT *S*. *aureus* USA300. Total CFUs in (D) blood, (E) heart and (F) lung of Fibγ^WT^ and Fibγ^Δ5^ mice 4 hours after i.p. infection with 4x10^8^ CFUs of WT *S*. *aureus* USA300. Data are represented as mean ± SEM and statistical significance was determined by Mann-Whitney u-test.

We also sought to determine whether the early, local failure in bacterial clearance displayed by Fibγ^Δ5^ mice was associated with enhanced bacterial dissemination. At 1 hour after peritoneal infection, the *S*. *aureus* burden was significantly higher in the blood as well as heart and lung (*e*.*g*., organs distant from the peritoneal cavity) of Fibγ^Δ5^ mice compared to Fibγ^WT^ mice (Fig 4A, 4B and 4C). At 4 hours after infection, higher levels of CFUs in blood, heart, and lung tissues were also observed in Fibγ^Δ5^ mice relative to Fibγ^WT^ mice (Fig 4D, 4E and 4F). To determine if the increased overall bacterial burden observed in Fibγ^Δ5^ mice resulted in systemic evidence of tissue damage to the host at the 4-hour time point, markers of coagulation activation and tissue damage were analyzed. There were no significant differences in plasma thrombin-antithrombin complexes and D-dimer in mice of any genotype (S1A and S1B Fig). No differences in plasma lactate dehydrogenase (LDH), creatine kinase (CK), or alanine aminotransferase (ALT) were detected based on genotype or infection (S1C, S1D and S1E Fig). A modest but statistically significant difference in cardiac troponin I was detected in infected Fibγ^Δ5^ mice relative to infected Fibγ^WT^ mice, consistent with the higher *S*. *aureus* bacterial burdens in the hearts of those animals (S1F Fig).

### Elimination of platelets but not platelet-derived protease-activated receptor-4 results in increased retrievable *S. aureus* in the peritoneal cavity

**Fig 5:**
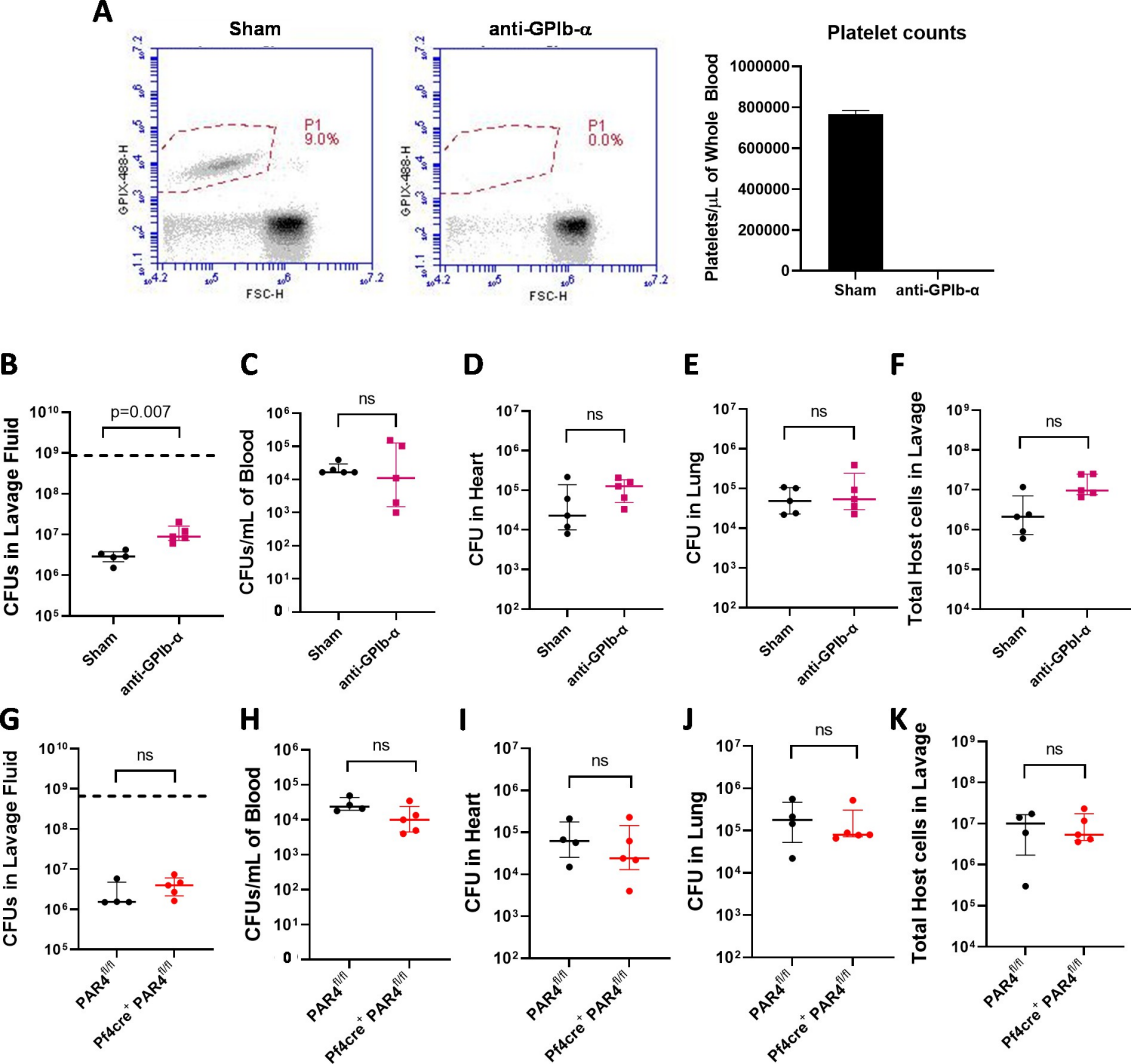
Platelets but not platelet-derived protease-activated receptor-4 are important for local *S. aureus* clearance from the peritoneal cavity. (A) Flow-cytometry analysis of platelet depletion following i.v. injection with anti-GPIb-α antibodies. Total bacterial CFUs in the peritoneal (B) lavage fluid, (C) blood, (D) heart and (E) lung of sham injected and platelet-depleted mice 1 hour after i.p. infection with 8x10^8^ CFUs of WT *S*. *aureus* USA300. (F) Total cell counts from peritoneal lavage fluid of sham injected and platelet-depleted mice 1 hour after infection with WT *S*. *aureus* USA300. Total bacteria CFUs in the peritoneal lavage (G) fluid, (H) blood, (I) heart and (J) lung of PAR4^fl/fl^ and Pf4-cre^+^/PAR4^fl/fl^ mice 1hr after i.p. infection with 6.57x10^8^ CFUs of WT *S*. *aureus* USA300. (K) Total cell counts from peritoneal lavage fluid of PAR4^fl/fl^ and Pf4-Cre^+^/PAR4^fl/fl^ mice 1 hour after infection with WT *S*. *aureus* USA300. Dashed horizontal lines indicate the infection dose. Data are presented as mean ± SEM and statistical significance was determined by Mann-Whitney u-test.

In addition to supporting binding to various *S*. *aureus* fibrinogen binding proteins, the fibrinogen γ-chain AGDV motif also supports binding to platelet integrin α_IIb_β_3_. Thus, differences in *S*. *aureus* infection observed in the Fibγ^Δ5^ mice could be linked to platelets or platelet function. To determine a potential contribution of platelets in *S*. *aureus* peritonitis, mice that received a sham injection or a platelet-depleting antibody (Fig 5A) were challenged for 1 hour with 8x10^8^ CFUs of WT *S*. *aureus* USA300 by intraperitoneal injection. Platelet-depleted mice showed a modest but significant increase in *S*. *aureus* CFUs in peritoneal lavage fluid (Fig 5B). However, no significant differences were observed in CFUs quantified from blood (Fig 5C), heart (Fig 5D) and lung (Fig 5E). Notably, there were no differences in the fibrinogen accumulation in the lavage fluid (S2A Fig) nor plasma fibrinogen levels between sham-treated and platelet-depleted mice (S2B Fig). The total number of host leukocytes retrievable from the peritoneal cavity were similar between both groups (Fig 5F).

Given that thrombin activity was previously documented as a component of the mechanism of fibrin-dependent *S*. *aureus* clearance from the peritoneal cavity [28], we next determined if protease-activated receptor-4 (PAR-4) mediated platelet activation was linked to changes in *S*. *aureus* clearance following peritoneal infection. Mice with a platelet-specific deletion in PAR-4 (i.e., Pf4-Cre/PAR4^fl/fl^) were analyzed. Pf4-Cre^+^/PAR4^fl/fl^ and PAR4^fl/fl^ controls were challenged for 1 hour with 6.6x10^8^ CFUs of WT *S*. *aureus* USA300 via i.p. injection. There were no observable differences in *S*. *aureus* CFUs in peritoneal lavage fluid (Fig 5G), blood (Fig 5H), heart (Fig 5I) and lung (Fig 5J). The total number of host leukocytes retrievable from the peritoneal cavity were also similar between both groups (Fig 5K). Collectively, these data suggest that platelets make a modest, but statistically significant, PAR4-independent contribution to the local host antimicrobial response against *S*. *aureus* peritonitis but elimination of platelets does not play a major role in promoting bacterial dissemination.

### Deletion of ClfA from *S. aureus* USA300 results in a reduction of bacterial clearance with an increased initial, but not sustained dissemination/accumulation of bacteria in distant organs

**Fig 6:**
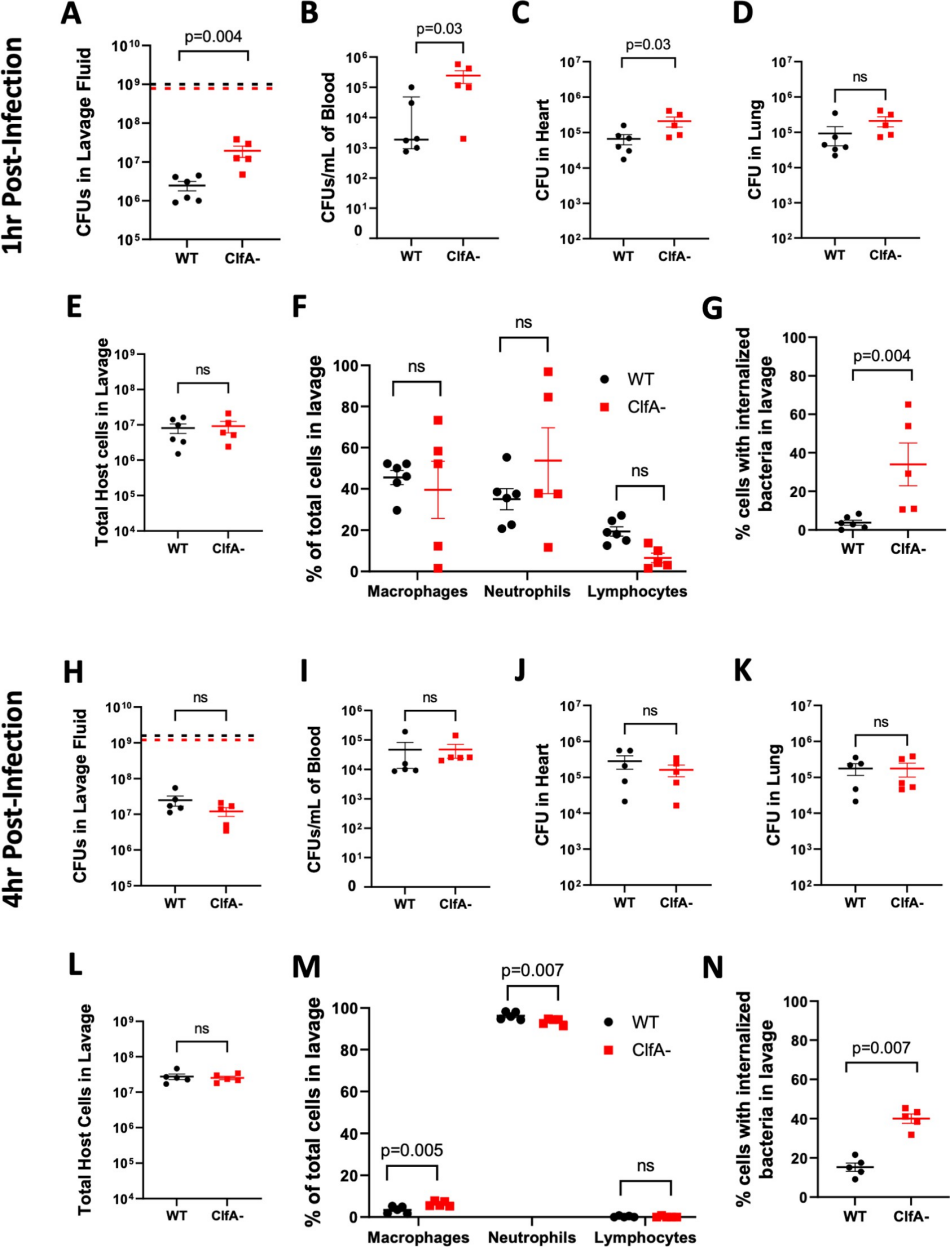
Deletion of ClfA from *S. aureus* USA300 results in increased *S. aureus* CFUs in the peritoneal cavity and a transient increase in CFUs the bloodstream and distant organs. Total live bacteria in (A) lavage, (B) blood, (C) heart and (D) Lung of WT mice 1 hour after i.p. infection with WT (1x10^9^ CFUs) or ClfA- (7.8x10^8^ CFUs) *S*. *aureus* USA300. (E) Total host cells, (F) host cell differentials, and (G) and percentage of host cells with internalized bacteria in lavage fluid at 1 hour after infection. Total live bacteria in (H) lavage, (I) blood, (J) heart and (K) lung of WT mice 4 hours after i.p. infection with WT (1.6x10^9^ CFUs) or ClfA- (1.2x10^9^ CFUs) *S*. *aureus* USA300. (L) Total host cells, (M) host cell differentials, and (N) and percentage of host cells with internalized bacteria in lavage fluid at 1 hour after infection. CFU data are presented as mean ± SEM and statistical significance was determined by Mann-Whitney u-test. Host cell counts and percentages are presented as mean ± SEM with statistical significance determined by 2-way ANOVA with Šídák’s multiple comparisons test. Data on cells with internalized bacteria are presented as mean ± SEM and statistical significance was determined by Mann-Whitney U-test. Dashed horizontal lines indicate the infection dose.

Our *in vitro* findings indicated that ClfA plays a major role in *S*. *aureus* adhesion and clumping to fibrinogen. To determine if ClfA is also critical for the host antimicrobial response and suppression of dissemination following peritoneal infection, WT mice were infected with WT or ClfA- *S*. *aureus* USA300 by intraperitoneal injection. Similar to what was observed in Fibγ^Δ5^ mice, mice challenged with ClfA- *S*. *aureus* USA300 had increased bacterial CFUs in the peritoneal cavity 1 hour after infection relative to mice infected with WT *S*. *aureus* (Fig 6A). Notably, the accumulation of fibrinogen within the peritoneal cavity was equivalently increased in mice infected with either WT or ClfA- *S*. *aureus* USA300 compared to uninfected mice (S2C Fig) with no significant changes in circulating fibrinogen (S2D Fig). In addition, mice infected with ClfA- *S*. *aureus* USA300 displayed a modest, but statistically significant increase in bacterial dissemination/accumulation into the blood (Fig 6B), heart (Fig 6C) and a trend to higher CFUs in the lung tissue (Fig 6D) compared to mice infected with WT *S*. *aureus*. Mice infected with WT or ClfA- *S*. *aureus* showed similar numbers of host leukocytes retrievable from the peritoneal cavity 1 hour after infection (Fig 6E). Differential cell counts from peritoneal lavage fluid revealed no significant differences in immune cell types in mice infected with WT or ClfA- *S*. *aureus* (Fig 6F). However, WT animals infected with ClfA- *S*. *aureus* showed higher numbers of host cells with internalized bacteria 1 hour after infection (Fig 6G). Notably, infection of WT mice with ClfA-^*pclfa*^ *S*. *aureus* resulted in local retrievable CFUs (S3A Fig) and disseminated bacteria (S3B–S3D Fig) in numbers virtually identical to WT *S*. *aureus*. Furthermore, host immune cell numbers (S3E Fig) were similar to mice infected for 1 hour with WT *S*. *aureus*. Collectively, these findings suggest that differences in local and systemic CFUs in mice infected with ClfA- *S*. *aureus* were due to the loss of ClfA itself and not to unintended secondary changes in the ClfA- *S*. *aureus* USA300 strain.

WT mice were also infected with WT or ClfA- *S*. *aureus* USA300 by intraperitoneal injection for 4 hours. Here, no differences in CFUs in peritoneal lavage fluid were observed between mice infected with WT or ClfA- *S*. *aureus*, consistent with what was observed in the Fibγ^Δ5^ mice (Fig 6H). However, while Fibγ^Δ5^ mice showed increased bacterial burdens in blood and organ tissues 4 hours after infection, WT mice infected with ClfA- *S*. *aureus* had similar CFUs in blood, heart, and lung tissue relative to mice infected with WT *S*. *aureus* (Fig 6I–6K). At this time point, no difference in total retrievable cells from the peritoneal cavity were found (Fig 6L). A modest, but statistically significant, difference in immune cell types was observed where mice infected with ClfA- *S*. *aureus* showed higher macrophage and lower neutrophil numbers when compared to mice infected with WT *S*. *aureus* (Fig 6M). Similar to findings at 1 hour, WT animals infected with ClfA- *S*. *aureus* had higher numbers of host cells with internalized bacteria at 4 hours after infection (Fig 6N). Collectively, these findings suggest that ClfA-fibrinogen binding significantly contributes to the acute host antimicrobial response in the peritoneal cavity and early dissemination. However, eliminating ClfA does not result in a sustained increase in bacterial accumulation within distant organs.

### Fibrin-macrophage driven host antimicrobial function limits dissemination, but fibrin matrix formation alone is not sufficient to suppress systemic spread of *S. aureus* from the peritoneal cavity

**Fig 7:**
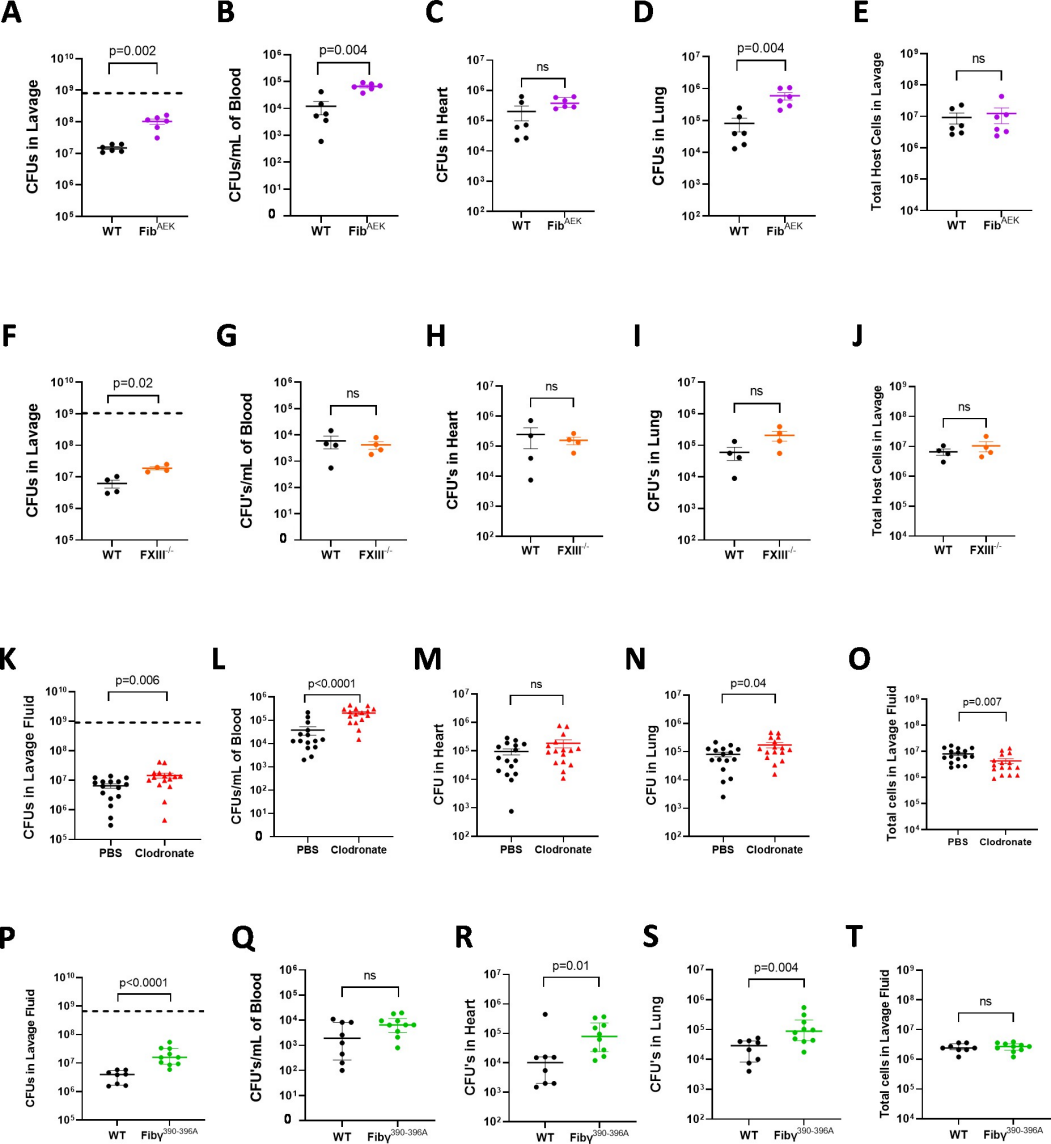
Fibrin-macrophage driven host antimicrobial function limits dissemination, but fibrin matrix formation alone is not sufficient to suppress systemic spread of *S. aureus* out of the peritoneal cavity. Total live bacteria in (A) lavage fluid, (B) blood, (C) heart and (D) lung and total host cell counts (E) from WT and Fib^AEK^ mice 1 hr after infection with 8.8x10^8^ CFUs of WT *S*. *aureus* USA300. Total live bacteria in lavage fluid (F), blood (G), heart (H) and lung (I) and total host cell counts (J) from WT and FXIII^-/-^ mice 1 hr after infection with 1x10^9^ CFUs of WT *S*. *aureus* USA300. Total live bacteria in lavage fluid (K), blood (L), heart (M) lung (N) and total host cell count (O) from WT mice treated with PBS or Clodronate liposomes 1 hr after infection with 9x10^8^ CFUs of WT *S*. *aureus* USA300. Total live bacteria in lavage fluid (P), blood (Q), heart (R) and lung (S) and total host cell counts (T) from WT and Fibγ^390-396A^ mice 1 hr after infection with 6.7x10^8^ CFUs of WT *S*. *aureus* USA300. Data are presented as mean ± SEM and statistical significance was determined by Mann-Whitney u-test.

To assess whether fibrin(ogen) is sufficient for limiting bacterial dissemination, Fib^AEK^ mice that express fibrinogen with a mutated Aα chain that renders it insensitive to thrombin cleavage and downstream polymer formation were infected with 8.8x10^8^ CFUs of WT *S*. *aureus* USA300 for 1 hour. As previously observed [29], Fib^AEK^ mice had significantly higher CFUs in peritoneal lavage fluid when compared to Fib^WT^ mice (Fig 7A). Notably, Fib^AEK^ mice also showed a significant increase in the bacterial CFUs in blood and lung, with a trend towards higher CFUs in the heart (Fig 7B, 7C and 7D). Total host cells retrieved in lavage showed similar numbers in WT and Fib^AEK^ mice (Fig 7E). Here, we also show for the first time that the fibrin crosslinking by transglutaminase Factor XIII (FXIII) contributes to host local antimicrobial function in the peritoneal cavity. Mice deficient in the catalytic A subunit of FXIII (*i*.*e*., F13a^-/-^ mice) had significantly higher CFUs in peritoneal lavage when compared to WT mice 1 hour after intraperitoneal infection with 1x10^9^ CFUs of WT *S*. *aureus* USA300 (Fig 7F). However, there were no differences in bacterial dissemination to the blood, heart, or lung (Fig 7G, 7H and 7I). There were also similar numbers of host cells retrieved from lavage in WT and F13a^-/-^ mice (Fig 7J). Collectively, these data indicate that both fibrin polymer formation and FXIIIa-crosslinking play important roles in the acute host antimicrobial response, but whereas loss of polymer formation enhances dissemination, loss of FXIII crosslinking does not increase *S*. *aureus* escape from the peritoneal cavity.

Finally, to ascertain whether fibrin formation in and of itself offers protection to the host by retaining bacteria within the peritoneal cavity and thus preventing dissemination, macrophage depletion studies were performed. Previous studies revealed that macrophages play an important role in eliminating *S*. *aureus* within the peritoneal cavity [19,30]. WT mice were given PBS control or Clodronate liposomes 24 hours before infection with 9x10^8^ CFUs of WT *S*. *aureus* USA300. Consistent with published data, macrophage depletion resulted in a reduction of bacterial clearance from the peritoneal cavity 1 hour after infection (Fig 7K). Here, we show that macrophage depletion also resulted in significantly increased bacterial dissemination into circulation (Fig 7L) and lung tissue (Fig 7N) and trends towards higher CFUs in heart tissue (Fig 7M). As expected, there was a reduction in retrievable total host cells in clodronate-treated mice (Fig 7O). To extend these findings, Fibγ^390-396A^ mice with a mutation in the fibrinogen γ chain that eliminates the leukocyte integrin α_M_β_2_ binding motif were analyzed. Previous studies documented that fibrinogen γ^390-396A^ polymerizes identical to WT fibrinogen but does not support fibrin-mediated macrophage binding and activation [31]. Following infection with 6.7x10^8^ CFUs of WT *S*. *aureus* USA300, Fibγ^390-396A^ mice displayed significantly increased CFUs in peritoneal lavage (Fig 7P). Importantly, Fibγ^390-396A^ mice also displayed a trend towards increased CFUs in the blood (Fig 7Q) and significantly increased CFUs in the heart and lung (Fig 7R and 7S, respectively), relative to infected WT mice. Analysis of total host cells in the peritoneal cavity showed no differences between WT and Fibγ^390-396A^ mice, again suggesting that the observed differences in bacterial clearance and dissemination were not due to changes in leukocyte numbers in the peritoneal cavity (Fig 7T). Collectively, these data suggest that fibrin polymer formation in the absence of the antimicrobial immune response is not sufficient for suppressing *S*. *aureus* dissemination following peritoneal infection.

## Discussion

Fibrin(ogen) is a centerpiece and trigger of a potent host *S*. *aureus* killing mechanism in the peritoneal cavity. Previous studies showed that whereas WT mice are able to eliminate ~99% of an initial peritoneal *S*. *aureus* infection within 15 minutes, Fib^-/-^ mice fail to eliminate the bacteria and rapidly succumb to the infection [19]. Here, we provide evidence that fibrin(ogen) binding to the microbe itself is a key component of the host bacterial killing mechanism. Elimination of the fibrinogen carboxy-terminal γ-chain AGDV motif (i.e., using Fibγ^Δ5^ mice) compromised bacterial killing. The number of retrievable *S*. *aureus* CFUs from the peritoneal cavity as well as the number of Fibγ^Δ5^ mice that succumbed to peritoneal *S*. *aureus* infection was significantly higher than those observed in WT mice, suggesting that elimination of *S*. *aureus*-fibrin(ogen) binding reduces the efficiency of the antimicrobial response. *S*. *aureus-*fibrin(ogen) binding was important for suppression of bacterial dissemination from the peritoneal cavity. Specifically, Fibγ^Δ5^ mice displayed higher levels of local *S*. *aureus* CFUs following infection, and robust levels of *S*. *aureus* in the blood and distant organ systems at 1 and 4 hours after peritoneal infection. Previous studies indicated that bacterial colonization into organs is accompanied by elevated markers of tissue damage in circulation at later time points after infection (*e*.*g*., 24 and 48 hours) [11]. Here, analyses at 4 hours after infection showed a modest, but statistically significant increase in cardiac troponin I was detected in Fibγ^Δ5^ mice. A finding that suggests cardiac activity may be particularly sensitive to the systemic spread of *S*. *aureus* microbes and that loss of heart function may be the basis for the increased mortality observed in Fibγ^Δ5^ mice.

The γ-chain AGDV motif is important for the binding of *S*. *aureus* to fibrin(ogen), but it is also a ligand for the platelet integrin α_IIb_β_3_ which plays a pivotal role in platelet aggregation [32,33]. Therefore, Fibγ^Δ5^ mice cannot effectively support fibrinogen-dependent platelet aggregation, but these animals retain normal platelet counts, plasma fibrinogen levels, clotting time, and fibrin crosslinking [27]. We found that platelet-depleted mice showed a modest, but significant, elevation in bacterial CFUs in the peritoneal lavage fluid compared to control mice. This finding is consistent with work from others showing that platelets contribute to host protection from *S*. *aureus* infection [34–36]. Additionally, clinical data has shown that patients that were thrombocytopenic at the onset of *S*. *aureus* bacteremia presented more commonly with severe sepsis that was accompanied by septic shock and renal failure [37]. Notably, the number of *S*. *aureus* CFUs present in the peritoneal cavity of Fibγ^Δ5^ mice (see Fig 3A) is higher than what is observed in both platelet-depleted mice (see Fig 5B) and mice infected with ClfA- *S*. *aureus* USA300 (see Fig 6A), suggesting that bacteria-fibrin(ogen) and platelet-fibrin(ogen) interactions may additively contribute to the host antimicrobial response to *S*. *aureus* peritonitis. The platelet contribution seems to be independent of thrombin-PAR4-mediated activation, as mice lacking PAR4 on platelets showed similar bacterial CFU numbers in peritoneal lavage fluid, blood, and distant organs when compared to WT mice. This finding is consistent with previous studies showing that thrombin activity in the peritoneal cavity following *S*. *aureus* infection does not occur through host tissue factor, but through *S*. *aureus* coagulase function [19], and that *S*. *aureus* coagulase-prothrombin complexes (staphylothrombin) do not activate platelets directly [38].

WT mice infected with *S*. *aureus* deficient in ClfA showed a similar phenotype where there was significantly compromised bacterial clearance. However, whereas loss of the fibrinogen γ-chain AGDV motif also resulted in enhanced bacterial dissemination and accumulation of microbes in distant organs, loss of ClfA had only a minor impact on these aspects of the infection. We speculate that the increased CFUs of WT *S*. *aureus* USA300 observed in blood, heart, and lung of Fibγ^Δ5^ mice are a result of the loss of interactions between the fibrinogen γ-chain AGDV motif and *S*. *aureus* factors other than ClfA. To this end, fibronectin-binding proteins Fnbp-A and Fnbp-B have been shown to bind this same region of fibrinogen [6]. It is possible that loss of fibrinogen binding with one or both of these *S*. *aureus* receptors, either alone or in combination with loss of ClfA-fibrin(ogen) binding, is the basis of increased CFUs in blood, heart, and lungs in Fibγ^Δ5^ mice. Intriguingly, our findings are inconsistent with the concept that *S*. *aureus* produces ClfA to function as a potent virulence factor. One explanation is that ClfA plays a beneficial role for *S*. *aureus* in the context of certain types of infections or host microenvironments and that this outweighs the detrimental role it plays in peritonitis. For example, in a mouse model of bacteremia, intravenous infection with ClfA- *S*. *aureus* resulted in less host lethality than WT *S*. *aureus* [11]. *S*. *aureus* lacking ClfA have also been shown to be less virulent in mouse models of septic arthritis and endocarditis where the phenotypes have been attributed to loss of binding to fibrinogen deposited in inflamed joint tissue or damaged heart valves [7,39]. Further, ClfA has been shown to exacerbate *S*. *aureus* and formation of organ abscesses that shield the bacteria from host antimicrobial mechanisms and allow for proliferation [40]. Thus, the host compartment appears to be a significant determinant of bacteria virulence factor function.

Fibrin polymer formation is an integral component of the host antimicrobial mechanism as shown by studies performed using Fib^AEK^ mice. Fib^AEK^ mice possess a mutated form of fibrinogen that is not susceptible to proteolytic cleavage by thrombin or *S*. *aureus* coagulase-prothrombin complexes (i.e., staphylothrombin) [29]. Fib^AEK^ mice displayed significantly compromised bacterial clearance followed by enhanced dissemination from the peritoneal cavity following *S*. *aureus* infection (see Fig 7) [29]. Macrophages are key effector cells in the host antimicrobial response to *S*. *aureus* peritonitis [19,30,31], and engage fibrinogen via the leukocyte integrin receptor α_M_β_2_. The α_M_β_2_ binding motif is cryptic and is exposed by the conformational change that occurs following cleavage of fibrinogen to fibrin [41,42], consistent with the requirement of fibrin polymer formation for the antimicrobial response. Selectively eliminating fibrin-macrophage binding (using Fibγ^390-396A^ mice) or macrophages themselves (using clodronate) resulted in a reduction in bacterial clearance from the lavage fluid and increased dissemination. Under these conditions, fibrinogen still accumulates in the peritoneal cavity and fibrin formation and *S*. *aureus* binding to fibrin(ogen) can still take place. Our findings suggest, fibrin matrix formation itself does not appear sufficient to retain the microbes in the peritoneal cavity. Whereas macrophage function is critical to the host antimicrobial response, the failure of bacterial killing observed in Fibγ^Δ5^ mice was not linked to compromised phagocytosis as a significant increase in the number of bacteria internalized within host cells of the peritoneal cavity was observed. This increase in bacterial phagocytosis could serve as a ‘second hit’ to the host as it has been shown that once *S*. *aureus* are internalized within host cells, they suppress intracellular killing mechanisms and are shielded from other host driven antimicrobial pathways which could result in a chronic infection [43–45]. Collectively, these data suggest the fibrin(ogen)-driven antimicrobial mechanism in the peritoneal cavity involves a tripartite complex of *S*. *aureus*-fibrin-macrophages (S4 Fig).

In this study, we show for the first time that the transglutaminase FXIII plays an important role in the host antimicrobial response against *S*. *aureus* peritonitis. *F13a*^*-/-*^ mice show a modest but significant increase in *S*. *aureus* CFUs retrieved from the peritoneal cavity compared to WT mice following infection, suggesting a diminished host antimicrobial response. Deicke, et al. showed that FXIII enhances entrapment of *Streptococcus pyogenes* by crosslinking the bacteria to fibrin and that elimination of FXIII resulted in increased bacterial dissemination and poor survival in a mouse model of skin and soft tissue infection [46]. Other studies suggest FXIII can crosslink fibrin to the surface of bacteria, including *S*. *aureus* and *E*. *coli*, leading to their sequestration in clots [47]. Thus, a possible mechanism by which FXIII could enhance bacterial clearance is by preventing *S*. *aureus* from escaping fibrin matrices. Interestingly, while elimination of FXIII results in reduced clearance of *S*. *aureus* from the peritoneal cavity, we did not observe differences in bacterial CFUs in blood and distant organs when compared to WT mice. *S*. *aureus* produces a non-proteolytic activator of plasminogen called staphylokinase which allows the bacteria to escape entrapment by promoting fibrin degradation [6]. Fibrin that is not crosslinked by FXIII is more sensitive to degradation by plasmin [48], so it is possible that elimination of FXIII could allow *S*. *aureus* to more readily escape entrapment by proteolytically degrading fibrin matrices. Another possibility is that fibrin cross-linked by FXIII could function as a more efficient ligand for α_M_β_2_ compared to non-cross-linked fibrin, thus driving a more potent macrophage-driven antimicrobial response. Currently, it is unknown if FXIII cross-linking has any effect of leukocyte binding via α_M_β_2_. Further studies looking at binding of leukocytes to immobilized fibrinogen, fibrin and cross-linked fibrin would be necessary to understand these interactions.

The frequency of *S*. *aureus* as the causative agent in peritoneal infections is less pronounced than other *Staphylococcal* species (e.g., *S*. *epidermidis*). Rather, bacteria lacking the virulence factors that engage host fibrinogen and other clotting system components integral to *S*. *aureus* virulence in bloodstream and soft tissue infection are prevalent sources of peritoneal infection [49]. In the peritoneal cavity, it is the host that utilizes clotting system proteins and many of the same *S*. *aureus* virulence factors to propagate a potent, acute, antimicrobial response. Current and previous work [29] has identified a deleterious effect of the Fibγ^Δ5^ and Fib^AEK^ mutations on host mortality following *S*. *aureus* peritonitis. However, additional studies are required to define the impact of eliminating the fibrin(ogen)-macrophage response, platelets, or fibrin crosslinking on host mortality; definitively linking specific fibrin-macrophage functions to the precise killing mechanism(s) for *S*. *aureus*, and potentially other microbes; and identify additional cells and antimicrobial molecules required for the antimicrobial response in the peritoneal cavity.

## Materials and methods

### Ethics statement

The University of North Carolina at Chapel Hill Institutional Animal Care and Use Committee (UNC-IACUC) approved all studies of mice performed under protocol number 19–204.

#### Mice

WT, Fibγ ^Δ5^ [27], Fib^AEK^ [29], Fibγ^390-396A^ [31], FXIII^-/-^ [50] and Pf4-Cre^+^/PAR4^fl/fl^ [51] mice were used in these studies. For each experiment, sex- and age-matched (i.e., both males and females of at 8–12 weeks of age) on a C57Bl/6J background were analyzed. Control mice were littermates derived from each colony analyzed.

### Bacteria stains and growth conditions

WT and ClfA- *S*. *aureus* USA300 LAC were used in the studies conducted here. In addition, we used a complementation strain of ClfA- *S*. *aureus* USA300 LAC where the clfA gene and its 216bp upstream region were amplified from USA300 strain JE2 chromosomal DNA and inserted into plasmid pSK236 [52] by Gibson assembly, to yield plasmid pclfA. The plasmid insert was verified by sequencing prior to transformation into RN4220 and subsequently the USA300 ClfA- mutant strain. Stationary phase bacteria were grown in tryptic soy broth (TSB) (BD Difco) at 37°C overnight, washed and re-suspended in phosphate-buffered solution (PBS), and diluted to an optical density (OD) at 600nm of 0.4, 1.0 or 6.0 based on the experiment. Exponential phase bacteria were grown in the same conditions overnight. The following morning, a small amount of the overnight culture was added to fresh TSB at a 1:50 ratio and incubated at 37°C. Every 30 min, the OD_600_ was measured until a value of 0.6 was reached. Then, cultures were washed and re-suspended in PBS, and diluted to an OD_600_ of 0.4, 1.0 or 6.0 based on the experiment.

### Fibrinogen purification

Fibrinogen was purified from citrate-plasma isolated from naïve Fibγ^WT^ and Fibγ^Δ5^ mice by ammonium sulfate precipitation. Briefly, whole blood is collected in 1:10 volume of 0.105 M citrate from inferior vena cava exsanguination and plasma isolated by centrifugation. Ammonium sulfate was added to plasma up to a concentration of 25% saturation to precipitate the fibrinogen, which was ultimately re-suspended in dialysis buffer (150mM NaCl, 20mM Hepes and 5mM ε-amino-n-caproic acid) and dialyzed overnight using the same buffer to remove any remaining ammonium sulfate.

### Fibrinogen-*S. aureus* adhesion assay

NUNC 96-well plates (Thermo Fisher) were coated with 100μL of diluted purified mouse fibrinogen in buffer (15mM Na_2_HCO_3_, 35mM NaHCO_3_, 3.2mM NaN_3_) to concentrations ranging from 0.25–25μg/mL and incubated overnight at 4°C. Following incubation, plates were washed three times with 100μ/well of Wash Buffer (150mM NaCl and 0.01% Tween20) and blocked with 100μL/well of 1% BSA, 0.05% Tween 20 solution in PBS. Plates were then incubated for 1hr at 37°C and subsequently washed three times with 100μL/well of wash buffer. Afterwards, 100μL/well of *S*. *aureus* suspension was added to each well and subsequently incubated for 2hr at 37°C. Bacterial suspensions contained either WT, ClfA- or ClfA-^*pclfa*^ *S*. *aureus* USA300 grown to stationary or exponential growth phase re-suspended at an OD_600_ of 0.4. Plates were then washed 3 times with 100μ/well of wash buffer and fixed for 30 min with 25% formaldehyde solution. Once fixed, plates were washed once with 100μL/well of wash buffer and stained with 0.1% crystal violet for 30min. Stain was removed by washing plates 3 times with 100μL/well of wash buffer. Remaining stain was solubilized in 10% acetic acid and placed in a plate reader to quantify absorbance at 570nm.

### Fibrinogen-*S. aureus* clumping assay

Purified mouse fibrinogen was diluted in PBS to suspensions ranging from 0.25–25μg/mL and 50μL/well was placed in 96-well tissue culture plates (BD Falcon). Stationary or exponential phase cultures of WT or ClfA-, ClfA-^*pclfa*^ *S*. *aureus* USA300 were prepared as a suspension at an OD_600_ of 6.0. with 20μL/well of the bacterial suspension added to 96-well tissue culture plates. Plates were agitated using an orbital shaker for 5 min. Clumping was measured by reading light transmission at 570nm.

### Mouse model of *S. aureus* peritonitis

Bacterial suspensions at a concentration of ~1x10^9^ CFUs/mL of stationary phase of WT or ClfA- USA300 *S*. *aureus* were prepared from overnight cultures and 1mL was administered by intraperitoneal injection to mice. After 1 or 4hr, mice were anesthetized with a cocktail of ketamine, xylazine and acepromazine followed by peritoneal lavage with 5mL of PBS, IVC blood draw and removal of the heart and lung. Serial dilutions of the lavage fluid, blood and homogenates of heart and lung were plated on Tryptic Soy Agar in duplicate and incubated overnight at 37°C. Colony counts were performed for each sample and compared to the inoculum dose to determine bacterial clearance. For survival studies, 10 mice per group of Fibγ^WT^ and Fibγ^Δ5^, were administered an intraperitoneal injection of ~5x10^8^ CFUs of *S*. *aureus* and monitored for 7 days. Humane endpoints included the loss of up to 30% of the initial mouse body weight and/or reaching a moribund state.

### Platelet and macrophage depletion

Platelets were depleted by intravenous injection of 2μg/g of anti-GPIb-α antibody (clone R300, Emfret Analytics) 3 hours prior to i.p. infection with WT *S*. *aureus* USA300. Following cell depletion, infection experiments were performed as described. Cell depletion was confirmed by flow cytometry analysis on blood collected 1hr after infection and stained with anti GPIX-AF488 antibody (Emfret Analytics). Macrophages were depleted using clodronate liposomes (Encapsula Nano Sciences) administered by intraperitoneal injection 24 hours prior to infection. Macrophage depletion was confirmed using FACS analysis in which cells from peritoneal lavage fluid were stained using BV605 Rat Anti-Mouse F4/80 (BD), and LIVE/DEAD Fixable Violet Cell Stain Kit (Thermo Fisher) and analyzed on the Attune flow cytometer. Total host cell counts within the lavage fluid were determined using a hemocytometer. Cytospin preparations of lavage fluid were stained with Kwik-Diff (Thermo Fisher Shandon) to determine differential host cell counts. Total cell counts were determined using a hemocytometer. Following cell depletion, infection experiments were performed as described.

### Cytospin analysis

A 1:3 (100μL of lavage fluid + 200μL of PBS) dilution of peritoneal lavage fluid was prepared and 150uL of the solution was used for cytospin. Cytospin slides are let dry overnight and stained with Diff-Quick stain (methanol fixative, eosinophilic solution and basophilic solution). Stained slides were mounted using permount and imaged at 20 and 40x magnification. Quantification of peritoneal cells with internalized bacteria was performed by inspection of stained cytospin preparations under 20X magnification with at ~200 cells counted per sample. Representative images were captured at 40x.

### Fibrinogen ELISA and Plasma Markers of Tissue Damage

Fibrinogen levels in peritoneal lavage fluid and citrated plasma were determined using Mouse Fibrinogen ELISA Kit (ICL). Tissue damage markers were measured in citrated plasma. Cardiac troponin I levels were determined by ELISA using a high-density mouse cardiac troponin I kit (Life Diagnostics, Inc.). Lactate dehydrogenase and creatine kinase were determined using a colorimetric activity assay (BioAssay Systems). Plasma alanine aminotransferase (ALT) levels were determined using an enzyme assay kit (Labs Biotechnology).

## Supporting information

S1 Fig *S. aureus* dissemination precedes overt tissue damage or increased intravascular coagulation. Circulating markers of tissue damage and coagulation activity were analyzed from mouse plasmas 4 hr after I.P. *S*. *aureus* infection. (A) Thrombin-anti-thrombin (TAT) complexes and (B) D-dimer were measured in circulation as markers of coagulation activation. Circulating levels of (C) lactate dehydrogenase (LDH) and (D) creatine kinase (CK) levels were analyzed as markers of muscle injury. (E) Alanine aminotransferase (ALT) as a marker of liver injury. (F) Circulating levels of cardiac troponin I was analyzed to assess damage to heart tissue. Data are presented as mean ± SEM and statistical significance was determined by 2-way ANOVA with Šídák’s multiple comparisons test.
(TIF)

S2 Fig Mice infected with WT or ClfA- *S. aureus* show increased fibrinogen levels in lavage compared to uninfected mice. (A) Fibrinogen ELISA on lavage fluid from WT that received a sham injection or platelet depleting antibody and were infected with WT *S*. *aureus* USA300. (B) Fibrinogen ELISA on plasma from WT that received a sham injection or platelet-depleting GPI-α antibody and were infected with WT *S*. *aureus* USA300. (C) Fibrinogen ELISA on lavage fluid from WT mice that were uninfected or infected with WT or ClfA- USA300 *S*. *aureus*. (D) Fibrinogen ELISA on plasma from WT mice that were uninfected or infected with WT or ClfA- USA300 *S*. *aureus*. Data is presented as mean ± SEM and statistical significance was determined by One-way ANOVA with Tukey’s multiple comparisons test.
(TIF)

S3 Fig Re-expression of ClfA in ClfA- *S. aureus* USA300 restores the infection profile observed with WT *S. aureus* USA300 in WT mice. Total live bacteria in (A) lavage, (B) blood, (C) heart and (D) Lung, and (E) Total host cells in WT mice 1 hour after i.p. infection with WT (1.07x10^9^ CFUs), ClfA- (1.19x10^9^ CFUs), or ClfA-^*pclfa*^(1.14x10^9^ CFUs) *S*. *aureus* USA300. Data is presented as mean ± SEM and statistical significance was determined by One-way ANOVA with Tukey’s multiple comparisons test. Dashed horizontal lines indicate the infection doses.
(TIF)

S4 Fig Model of host antimicrobial response to *S. aureus* peritonitis. Following peritoneal *S*. *aureus* infection, fibrinogen enters the peritoneal cavity where it is converted to fibrin polymer by staphylothrombin and crosslinked by FXIIIa. Fibrin matrices bind both *S*. *aureus* and host macrophages via the γ-chain portion of the D-domain to drive a potent antimicrobial host defense response that kills the invading *S*. *aureus* and prevents dissemination. Platelets also contribute to the antimicrobial response but the mechanism is unknown.
(TIF)

## References

[1] Brown AF, Leech JM, Rogers TR, McLoughlin RM. Staphylococcus aureus Colonization: Modulation of Host Immune Response and Impact on Human Vaccine Design. Front Immunol. 2014;4:507. doi: 10.3389/fimmu.2013.00507 24409186 PMC3884195

[2] Lowy FD. Staphylococcus aureus infections. N Engl J Med. 1998;339(8):520–32. doi: 10.1056/NEJM199808203390806 9709046

[3] Tong SY, Davis JS, Eichenberger E, Holland TL, Fowler VG Jr. Staphylococcus aureus infections: epidemiology, pathophysiology, clinical manifestations, and management. Clin Microbiol Rev. 2015;28(3):603–61. doi: 10.1128/CMR.00134-14 26016486 PMC4451395

[4] Melzer M, Welch C. Thirty-day mortality in UK patients with community-onset and hospital-acquired meticillin-susceptible Staphylococcus aureus bacteraemia. J Hosp Infect. 2013;84(2):143–50. doi: 10.1016/j.jhin.2012.12.013 23602415

[5] Pottinger PS. Methicillin-resistant Staphylococcus aureus infections. Med Clin North Am. 2013;97(4):601–19, x. doi: 10.1016/j.mcna.2013.02.005 23809716

[6] Ko YP, Flick MJ. Fibrinogen Is at the Interface of Host Defense and Pathogen Virulence in Staphylococcus aureus Infection. Semin Thromb Hemost. 2016;42(4):408–21. doi: 10.1055/s-0036-1579635 27056151 PMC5514417

[7] Moreillon P, Entenza JM, Francioli P, McDevitt D, Foster TJ, Francois P, et al. Role of Staphylococcus aureus coagulase and clumping factor in pathogenesis of experimental endocarditis. Infect Immun. 1995;63(12):4738–43. doi: 10.1128/iai.63.12.4738-4743.1995 7591130 PMC173679

[8] Fei Y, Wang W, Kwiecinski J, Josefsson E, Pullerits R, Jonsson IM, et al. The combination of a tumor necrosis factor inhibitor and antibiotic alleviates staphylococcal arthritis and sepsis in mice. J Infect Dis. 2011;204(3):348–57. doi: 10.1093/infdis/jir266 21742832

[9] Que YA, Haefliger JA, Piroth L, Francois P, Widmer E, Entenza JM, et al. Fibrinogen and fibronectin binding cooperate for valve infection and invasion in Staphylococcus aureus experimental endocarditis. J Exp Med. 2005;201(10):1627–35. doi: 10.1084/jem.20050125 15897276 PMC2212930

[10] McAdow M, Kim HK, Dedent AC, Hendrickx AP, Schneewind O, Missiakas DM. Preventing Staphylococcus aureus sepsis through the inhibition of its agglutination in blood. PLoS Pathog. 2011;7(10):e1002307. doi: 10.1371/journal.ppat.1002307 22028651 PMC3197598

[11] Flick MJ, Du X, Prasad JM, Raghu H, Palumbo JS, Smeds E, et al. Genetic elimination of the binding motif on fibrinogen for the S. aureus virulence factor ClfA improves host survival in septicemia. Blood. 2013;121(10):1783–94. doi: 10.1182/blood-2012-09-453894 23299312 PMC3591798

[12] Negron O, Flick MJ. Does fibrinogen serve the host or the microbe in Staphylococcus infection? Curr Opin Hematol. 2019;26(5):343–8. doi: 10.1097/MOH.0000000000000527 31348048

[13] Macrae FL, Duval C, Papareddy P, Baker SR, Yuldasheva N, Kearney KJ, et al. A fibrin biofilm covers blood clots and protects from microbial invasion. J Clin Invest. 2018;128(8):3356–68. doi: 10.1172/JCI98734 29723163 PMC6063501

[14] Forsyth CB, Solovjov DA, Ugarova TP, Plow EF. Integrin alpha(M)beta(2)-mediated cell migration to fibrinogen and its recognition peptides. J Exp Med. 2001;193(10):1123–33. doi: 10.1084/jem.193.10.1123 11369784 PMC2193326

[15] Languino LR, Plescia J, Duperray A, Brian AA, Plow EF, Geltosky JE, et al. Fibrinogen mediates leukocyte adhesion to vascular endothelium through an ICAM-1-dependent pathway. Cell. 1993;73(7):1423–34. doi: 10.1016/0092-8674(93)90367-y 8100742

[16] Rubel C, Fernandez GC, Dran G, Bompadre MB, Isturiz MA, Palermo MS. Fibrinogen promotes neutrophil activation and delays apoptosis. J Immunol. 2001;166(3):2002–10. doi: 10.4049/jimmunol.166.3.2002 11160249

[17] Rubel C, Fernandez GC, Rosa FA, Gomez S, Bompadre MB, Coso OA, et al. Soluble fibrinogen modulates neutrophil functionality through the activation of an extracellular signal-regulated kinase-dependent pathway. J Immunol. 2002;168(7):3527–35. doi: 10.4049/jimmunol.168.7.3527 11907115

[18] Smiley ST, King JA, Hancock WW. Fibrinogen stimulates macrophage chemokine secretion through toll-like receptor 4. J Immunol. 2001;167(5):2887–94. doi: 10.4049/jimmunol.167.5.2887 11509636

[19] Prasad JM, Negron O, Du X, Mullins ES, Palumbo JS, Gilbertie JM, et al. Host fibrinogen drives antimicrobial function in Staphylococcus aureus peritonitis through bacterial-mediated prothrombin activation. Proc Natl Acad Sci U S A. 2021;118(1). doi: 10.1073/pnas.2009837118 33443167 PMC7817220

[20] Strong DD, Laudano AP, Hawiger J, Doolittle RF. Isolation, characterization, and synthesis of peptides from human fibrinogen that block the staphylococcal clumping reaction and construction of a synthetic clumping particle. Biochemistry. 1982;21(6):1414–20. doi: 10.1021/bi00535a048 7074096

[21] Ganesh VK, Rivera JJ, Smeds E, Ko YP, Bowden MG, Wann ER, et al. A structural model of the Staphylococcus aureus ClfA-fibrinogen interaction opens new avenues for the design of anti-staphylococcal therapeutics. PLoS Pathog. 2008;4(11):e1000226. doi: 10.1371/journal.ppat.1000226 19043557 PMC2582960

[22] Wann ER, Gurusiddappa S, Hook M. The fibronectin-binding MSCRAMM FnbpA of Staphylococcus aureus is a bifunctional protein that also binds to fibrinogen. J Biol Chem. 2000;275(18):13863–71. doi: 10.1074/jbc.275.18.13863 10788510

[23] Ni Eidhin D, Perkins S, Francois P, Vaudaux P, Hook M, Foster TJ. Clumping factor B (ClfB), a new surface-located fibrinogen-binding adhesin of Staphylococcus aureus. Mol Microbiol. 1998;30(2):245–57. doi: 10.1046/j.1365-2958.1998.01050.x 9791170

[24] Wolz C, Pohlmann-Dietze P, Steinhuber A, Chien YT, Manna A, van Wamel W, et al. Agr-independent regulation of fibronectin-binding protein(s) by the regulatory locus sar in Staphylococcus aureus. Mol Microbiol. 2000;36(1):230–43. doi: 10.1046/j.1365-2958.2000.01853.x 10760180

[25] Saravia-Otten P, Muller HP, Arvidson S. Transcription of Staphylococcus aureus fibronectin binding protein genes is negatively regulated by agr and an agr-independent mechanism. J Bacteriol. 1997;179(17):5259–63. doi: 10.1128/jb.179.17.5259-5263.1997 9286974 PMC179390

[26] Higgins J, Loughman A, van Kessel KP, van Strijp JA, Foster TJ. Clumping factor A of Staphylococcus aureus inhibits phagocytosis by human polymorphonuclear leucocytes. FEMS Microbiol Lett. 2006;258(2):290–6. doi: 10.1111/j.1574-6968.2006.00229.x 16640587

[27] Holmback K, Danton MJ, Suh TT, Daugherty CC, Degen JL. Impaired platelet aggregation and sustained bleeding in mice lacking the fibrinogen motif bound by integrin alpha IIb beta 3. EMBO J. 1996;15(21):5760–71. 8918453 PMC452323

[28] Mullins ES, Kombrinck KW, Talmage KE, Shaw MA, Witte DP, Ullman JM, et al. Genetic elimination of prothrombin in adult mice is not compatible with survival and results in spontaneous hemorrhagic events in both heart and brain. Blood. 2009;113(3):696–704. doi: 10.1182/blood-2008-07-169003 18927430 PMC2628376

[29] Prasad JM, Gorkun OV, Raghu H, Thornton S, Mullins ES, Palumbo JS, et al. Mice expressing a mutant form of fibrinogen that cannot support fibrin formation exhibit compromised antimicrobial host defense. Blood. 2015;126(17):2047–58. doi: 10.1182/blood-2015-04-639849 26228483 PMC4616238

[30] Brown AF, Murphy AG, Lalor SJ, Leech JM, O’Keeffe KM, Mac Aogain M, et al. Memory Th1 Cells Are Protective in Invasive Staphylococcus aureus Infection. PLoS Pathog. 2015;11(11):e1005226. doi: 10.1371/journal.ppat.1005226 26539822 PMC4634925

[31] Flick MJ, Du X, Witte DP, Jirouskova M, Soloviev DA, Busuttil SJ, et al. Leukocyte engagement of fibrin(ogen) via the integrin receptor alphaMbeta2/Mac-1 is critical for host inflammatory response in vivo. J Clin Invest. 2004;113(11):1596–606. doi: 10.1172/JCI20741 15173886 PMC419487

[32] Caen JP, Rosa JP. Platelet-vessel wall interaction: from the bedside to molecules. Thromb Haemost. 1995;74(1):18–24. 8578453

[33] Nurden AT, Nurden P. Congenital platelet disorders and understanding of platelet function. Br J Haematol. 2014;165(2):165–78. doi: 10.1111/bjh.12662 24286193

[34] Wuescher LM, Takashima A, Worth RG. A novel conditional platelet depletion mouse model reveals the importance of platelets in protection against Staphylococcus aureus bacteremia. J Thromb Haemost. 2015;13(2):303–13. doi: 10.1111/jth.12795 25418277 PMC4320667

[35] Yeaman MR, Norman DC, Bayer AS. Platelet microbicidal protein enhances antibiotic-induced killing of and postantibiotic effect in Staphylococcus aureus. Antimicrob Agents Chemother. 1992;36(8):1665–70. doi: 10.1128/AAC.36.8.1665 1416849 PMC192027

[36] Trier DA, Gank KD, Kupferwasser D, Yount NY, French WJ, Michelson AD, et al. Platelet antistaphylococcal responses occur through P2X1 and P2Y12 receptor-induced activation and kinocidin release. Infect Immun. 2008;76(12):5706–13. doi: 10.1128/IAI.00935-08 18824536 PMC2583569

[37] Gafter-Gvili A, Mansur N, Bivas A, Zemer-Wassercug N, Bishara J, Leibovici L, et al. Thrombocytopenia in Staphylococcus aureus bacteremia: risk factors and prognostic importance. Mayo Clin Proc. 2011;86(5):389–96. doi: 10.4065/mcp.2010.0705 21531882 PMC3084641

[38] Vanassche T, Kauskot A, Verhaegen J, Peetermans WE, van Ryn J, Schneewind O, et al. Fibrin formation by staphylothrombin facilitates Staphylococcus aureus-induced platelet aggregation. Thromb Haemost. 2012;107(6):1107–21. doi: 10.1160/TH11-12-0891 22437005

[39] Josefsson E, Hartford O, O’Brien L, Patti JM, Foster T. Protection against experimental Staphylococcus aureus arthritis by vaccination with clumping factor A, a novel virulence determinant. J Infect Dis. 2001;184(12):1572–80. doi: 10.1086/324430 11740733

[40] Cheng AG, Kim HK, Burts ML, Krausz T, Schneewind O, Missiakas DM. Genetic requirements for Staphylococcus aureus abscess formation and persistence in host tissues. FASEB J. 2009;23(10):3393–404. doi: 10.1096/fj.09-135467 19525403 PMC2747682

[41] Loike JD, Silverstein R, Wright SD, Weitz JI, Huang AJ, Silverstein SC. The role of protected extracellular compartments in interactions between leukocytes, and platelets, and fibrin/fibrinogen matrices. Ann N Y Acad Sci. 1992;667:163–72. doi: 10.1111/j.1749-6632.1992.tb51608.x 1309032

[42] Lishko VK, Kudryk B, Yakubenko VP, Yee VC, Ugarova TP. Regulated unmasking of the cryptic binding site for integrin alpha M beta 2 in the gamma C-domain of fibrinogen. Biochemistry. 2002;41(43):12942–51. doi: 10.1021/bi026324c 12390020

[43] Koziel J, Maciag-Gudowska A, Mikolajczyk T, Bzowska M, Sturdevant DE, Whitney AR, et al. Phagocytosis of Staphylococcus aureus by macrophages exerts cytoprotective effects manifested by the upregulation of antiapoptotic factors. PLoS One. 2009;4(4):e5210. doi: 10.1371/journal.pone.0005210 19381294 PMC2668171

[44] Jorch SK, Surewaard BG, Hossain M, Peiseler M, Deppermann C, Deng J, et al. Peritoneal GATA6+ macrophages function as a portal for Staphylococcus aureus dissemination. J Clin Invest. 2019;129(11):4643–56. doi: 10.1172/JCI127286 31545300 PMC6819137

[45] Neupane R, Bhatt N, Poudyal A, Sharma A. Methicillin-Resistant Staphylococcus Aureus Nasal Carriers among Laboratory Technical Staff of Tertiary Hospital in Eastern Nepal. Kathmandu Univ Med J (KUMJ). 2020;18(69):3–8. 33582679

[46] Deicke C, Chakrakodi B, Pils MC, Dickneite G, Johansson L, Medina E, et al. Local activation of coagulation factor XIII reduces systemic complications and improves the survival of mice after Streptococcus pyogenes M1 skin infection. Int J Med Microbiol. 2016;306(7):572–9. doi: 10.1016/j.ijmm.2016.06.001 27338836

[47] Wang Z, Wilhelmsson C, Hyrsl P, Loof TG, Dobes P, Klupp M, et al. Pathogen entrapment by transglutaminase—a conserved early innate immune mechanism. PLoS Pathog. 2010;6(2):e1000763. doi: 10.1371/journal.ppat.1000763 20169185 PMC2820530

[48] Rijken DC, Abdul S, Malfliet JJ, Leebeek FW, Uitte De Willige S. Compaction of fibrin clots reveals the antifibrinolytic effect of factor XIII: reply. J Thromb Haemost. 2017;15(1):205–6. doi: 10.1111/jth.13544 27748995

[49] Camargo CH, Cunha Mde L, Caramori JC, Mondelli AL, Montelli AC, Barretti P. Peritoneal dialysis-related peritonitis due to coagulase-negative Staphylococcus: a review of 115 cases in a Brazilian center. Clin J Am Soc Nephrol. 2014;9(6):1074–81. doi: 10.2215/CJN.09280913 24677560 PMC4046731

[50] Lauer P, Metzner HJ, Zettlmeissl G, Li M, Smith AG, Lathe R, et al. Targeted inactivation of the mouse locus encoding coagulation factor XIII-A: hemostatic abnormalities in mutant mice and characterization of the coagulation deficit. Thromb Haemost. 2002;88(6):967–74. 12529747

[51] Lee RH, Kawano T, Grover SP, Bharathi V, Martinez D, Cowley DO, et al. Genetic deletion of platelet PAR4 results in reduced thrombosis and impaired hemostatic plug stability. J Thromb Haemost. 2021. doi: 10.1111/jth.15569 34689407 PMC8792346

[52] Chien Y, Manna AC, Projan SJ, Cheung AL. SarA, a global regulator of virulence determinants in Staphylococcus aureus, binds to a conserved motif essential for sar-dependent gene regulation. J Biol Chem. 1999;274(52):37169–76. doi: 10.1074/jbc.274.52.37169 10601279

