# Supplementary figures and images for: Fibrin(ogen) engagement of S. aureus promotes the host antimicrobial response and suppression of microbe dissemination following peritoneal infection
Source: PLoS Pathog. 2022 Jan 18;18(1):e1010227. doi: 10.1371/journal.ppat.1010227 (PMC8797238; doi:10.1371/journal.ppat.1010227)

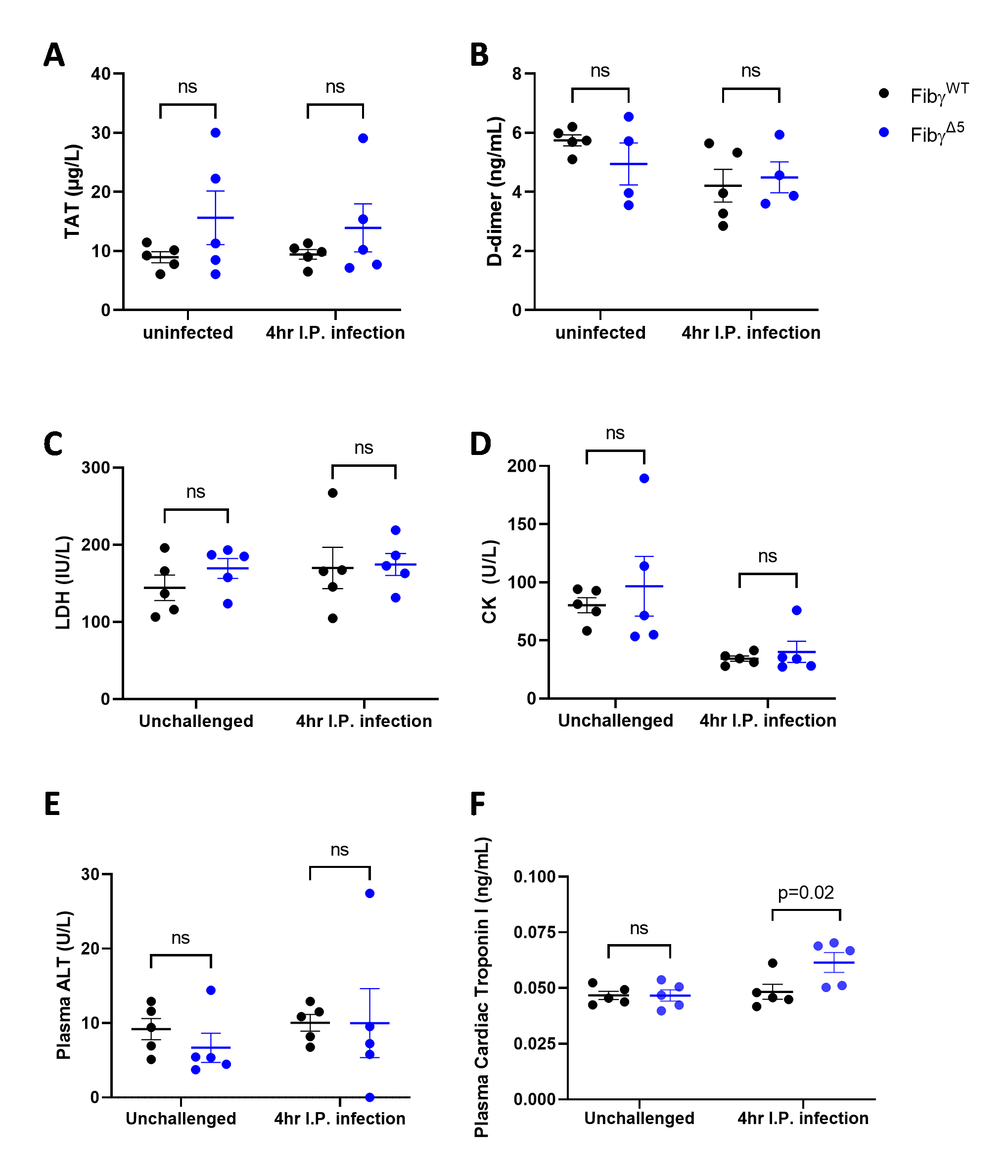

Supplement: S1 Fig — Circulating markers of tissue damage and coagulation activity were analyzed from mouse plasmas 4 hr after I.P. S. aureus infection. (A) Thrombin-anti-thrombin (TAT) complexes and (B) D-dimer were measured in circulation as markers of coagulation activation. Circulating levels of (C) lactate dehydrogenase (LDH) and (D) creatine kinase (CK) levels were analyzed as markers of muscle injury. (E) Alanine aminotransferase (ALT) as a marker of liver injury. (F) Circulating levels of cardiac troponin I was analyzed to assess damage to heart tissue. Data are presented as mean ± SEM and statistical significance was determined by 2-way ANOVA with Šídák’s multiple comparisons test. (TIF) [file ppat.1010227.s001.tif]

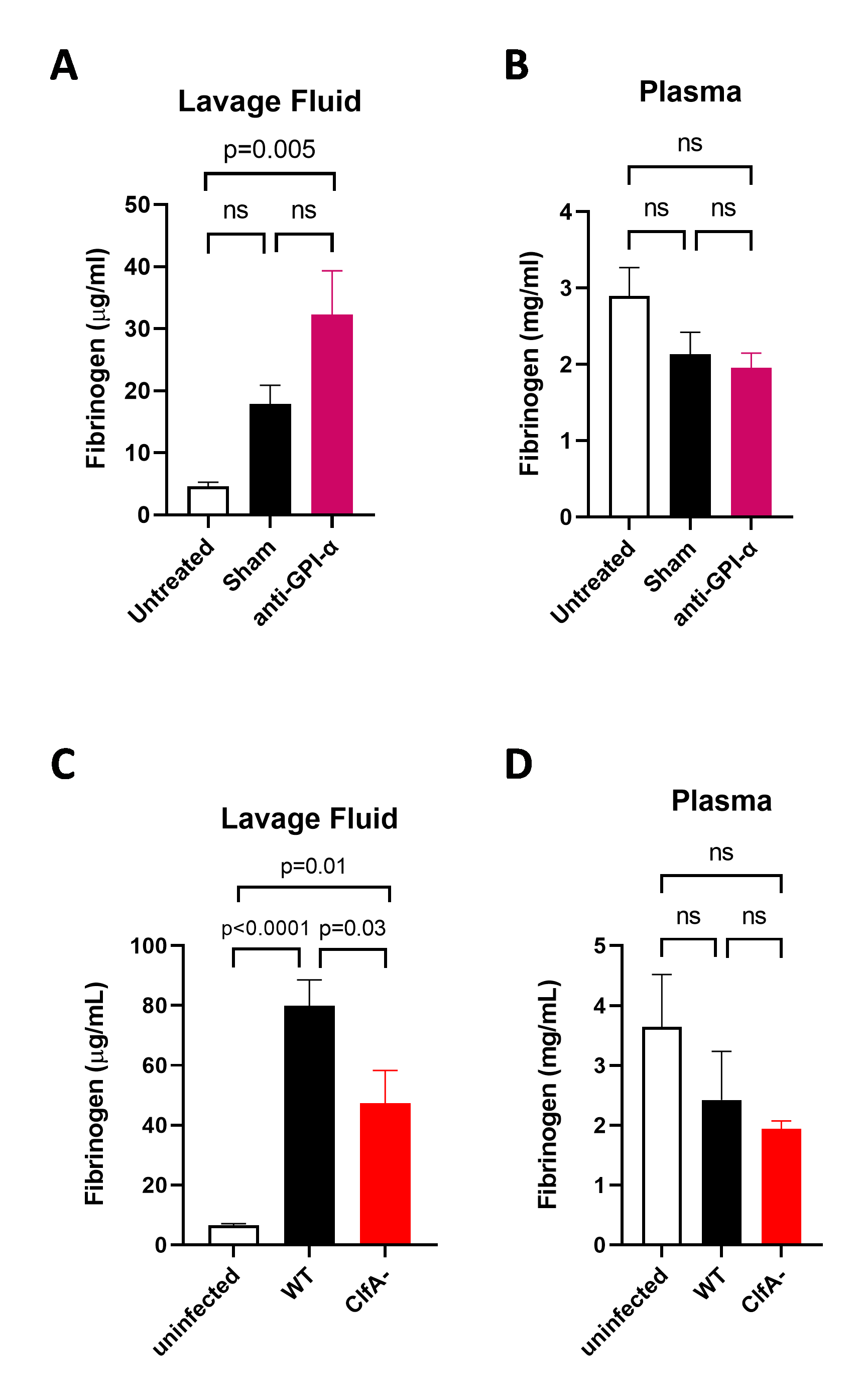

Supplement: S2 Fig — (A) Fibrinogen ELISA on lavage fluid from WT that received a sham injection or platelet depleting antibody and were infected with WT S. aureus USA300. (B) Fibrinogen ELISA on plasma from WT that received a sham injection or platelet-depleting GPI-α antibody and were infected with WT S. aureus USA300. (C) Fibrinogen ELISA on lavage fluid from WT mice that were uninfected or infected with WT or ClfA- USA300 S. aureus. (D) Fibrinogen ELISA on plasma from WT mice that were uninfected or infected with WT or ClfA- USA300 S. aureus. Data is presented as mean ± SEM and statistical significance was determined by One-way ANOVA with Tukey’s multiple comparisons test. (TIF) [file ppat.1010227.s002.tif]

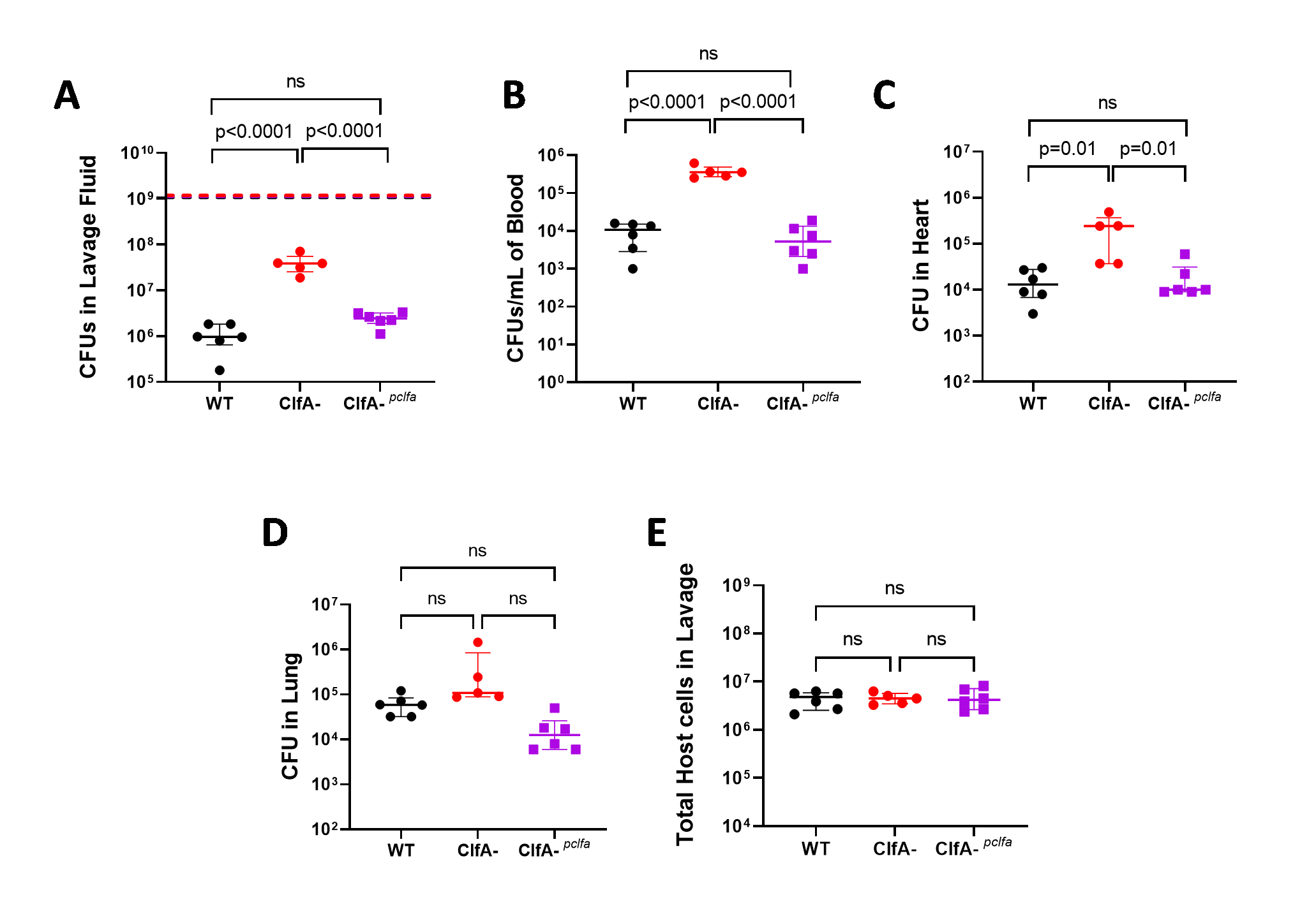

Supplement: S3 Fig — Total live bacteria in (A) lavage, (B) blood, (C) heart and (D) Lung, and (E) Total host cells in WT mice 1 hour after i.p. infection with WT (1.07x10^9^ CFUs), ClfA- (1.19x10^9^ CFUs), or ClfA-pclfa(1.14x10^9^ CFUs) S. aureus USA300. Data is presented as mean ± SEM and statistical significance was determined by One-way ANOVA with Tukey’s multiple comparisons test. Dashed horizontal lines indicate the infection doses. (TIF) [file ppat.1010227.s003.tif]

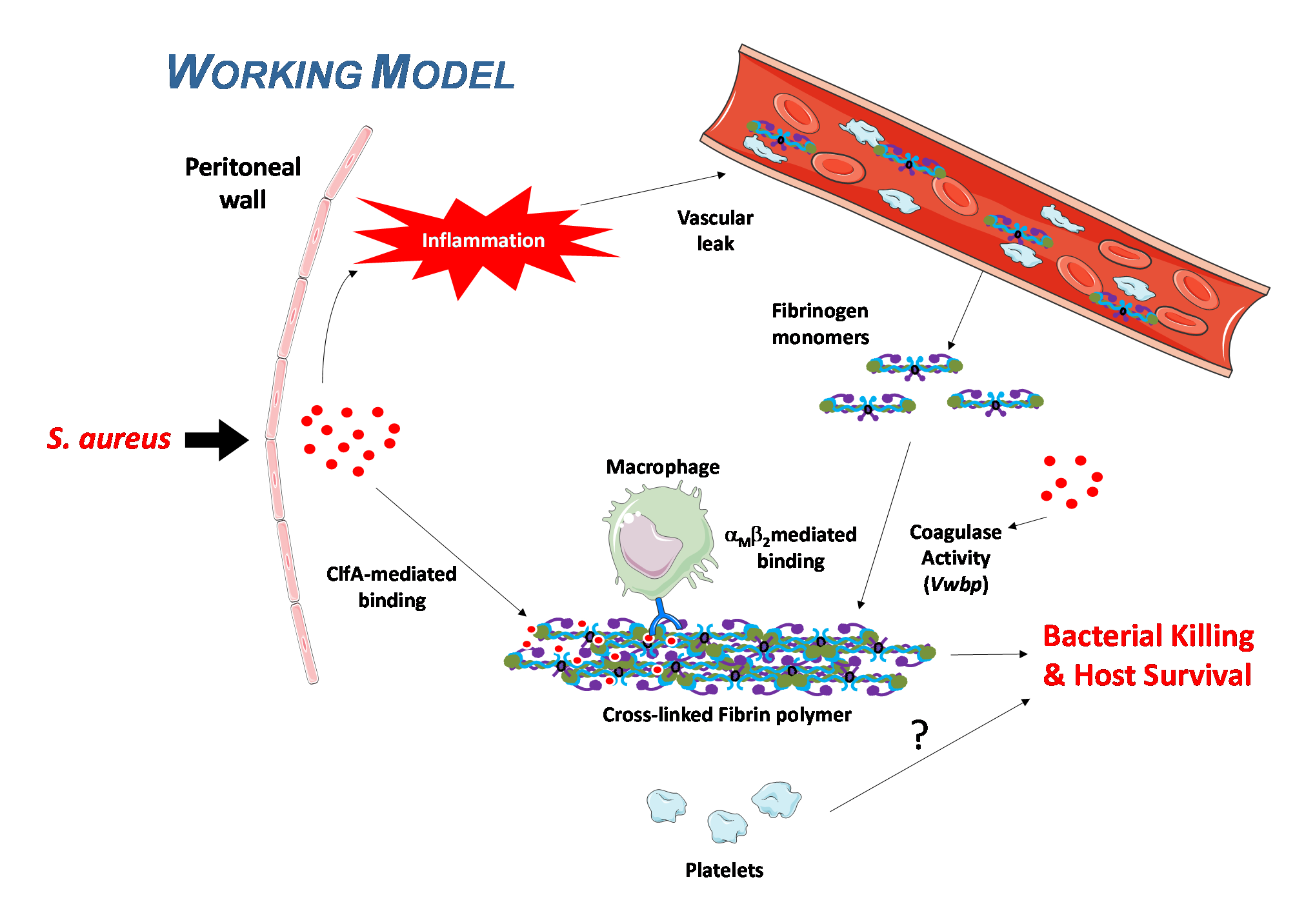

Supplement: S4 Fig — Following peritoneal S. aureus infection, fibrinogen enters the peritoneal cavity where it is converted to fibrin polymer by staphylothrombin and crosslinked by FXIIIa. Fibrin matrices bind both S. aureus and host macrophages via the γ-chain portion of the D-domain to drive a potent antimicrobial host defense response that kills the invading S. aureus and prevents dissemination. Platelets also contribute to the antimicrobial response but the mechanism is unknown. (TIF) [file ppat.1010227.s004.tif]
